# Spatial maps of hepatocellular carcinoma transcriptomes highlight an unexplored landscape of heterogeneity and a novel gene signature for survival

**DOI:** 10.1186/s12935-021-02430-9

**Published:** 2022-02-02

**Authors:** Nan Zhao, Yanhui Zhang, Runfen Cheng, Danfang Zhang, Fan Li, Yuhong Guo, Zhiqiang Qiu, Xueyi Dong, Xinchao Ban, Baocun Sun, Xiulan Zhao

**Affiliations:** 1 Department of Pathology, Tianjin Medical University, No. 22 Qixiangtai Road, Heping District, Tianjin, 300070 China; 2 Department of Pathology, Cancer Hospital of Tianjin Medical University, Tianjin, 300060 China; 3 Department of Pathology, General Hospital of Tianjin Medical University, Tianjin, 300052 China

**Keywords:** Hepatocellular carcinoma (HCC), Heterogeneity, Spatial transcriptomics (ST), Satellite nodules, Gene signature

## Abstract

**Background:**

Hepatocellular carcinoma (HCC) often presents with satellite nodules, rendering current curative treatments ineffective in many patients. The heterogeneity of HCC is a major challenge in personalized medicine. The emergence of spatial transcriptomics (ST) provides a powerful strategy for delineating the complex molecular landscapes of tumours.

**Methods:**

In this study, the heterogeneity of tissue-wide gene expression in tumour and adjacent nonneoplastic tissues using ST technology were investigated. The transcriptomes of nearly 10,820 tissue regions and identified the main gene expression clusters and their specific marker genes (differentially expressed genes, DEGs) in patients were analysed. The DEGs were analysed from two perspectives. First, two distinct gene profiles were identified to be associated with satellite nodules and conducted a more comprehensive analysis of both gene profiles. Their clinical relevance in human HCC was validated with Kaplan–Meier (KM) Plotter. Second, DEGs were screened with The Cancer Genome Atlas (TCGA) database to divide the HCC cohort into high- and low-risk groups according to Cox analysis. HCC patients from the International Cancer Genome Consortium (ICGC) cohort were used for validation. KM analysis was used to compare the overall survival (OS) between the high- and low-risk groups. Univariate and multivariate Cox analyses were applied to determine the independent predictors for OS.

**Results:**

Novel markers for the prediction of satellite nodules were identified and a tumour clusters-specific marker gene signature model (6 genes) for HCC prognosis was constructed.

**Conclusion:**

The establishment of marker gene profiles may be an important step towards an unbiased view of HCC, and the 6-gene signature can be used for prognostic prediction in HCC. This analysis will help us to clarify one of the possible sources of HCC heterogeneity and uncover pathogenic mechanisms and novel antitumour drug targets.

**Supplementary Information:**

The online version contains supplementary material available at 10.1186/s12935-021-02430-9.

## Introduction

Globally, the mortality rate of primary liver cancer ranks fourth among cancers. In many countries, its 5-year survival rate is less than 20%, and there have been no significant changes in survival rate over time [1–3]. Hepatocellular carcinoma (HCC) is the most common histological type accounting for the highest proportion (75–85%) of primary liver cancers [1]. Recently, progression has been considered to be the most important reason for the poor prognosis of patients with HCC, and identifying the possible factors affecting the progression of HCC and exploring potential interventional therapies will improve the prognosis of HCC patients [4].

HCC is an extraordinarily heterogeneous malignant disease considering the tumours that have thus far been identified. During personalized treatment of tumour patients, intra- and intertumour heterogeneity present a great challenge since they may directly change the predicted biological markers related to diagnosis, prognosis and therapy. Even for tumours with identical histological features, variation in the expression of biomarkers among different patients and between different tumour areas of the same individual sample, such as tumour and peritumour areas, should be considered seriously.

In HCC, the existence of microsatellite nodules is a well-known risk factor. However, such nodules usually cannot be detected on imaging modalities [5]. These satellite nodules undetectable by imaging may be risk factors for local recurrence since they are usually ignored by local ablation therapy. Therefore, analysing the risk factors associated with satellite nodules is very important to improve the treatment strategies for patients at high risk. Characterization of the tumour heterogeneity of HCC with satellite nodules using transcriptomic analysis may be important to reduce the incidence of local recurrence.

In this study, clusters defined by ST-related specific marker genes (differentially expressed genes; DEGs) were analysed from two perspectives. The feasibility and value of using ST to dissect inter- and intratumoural heterogeneity across HCC patient specimens with or without satellite nodules were demonstrated. In addition, a prognostic signature with DEGs were constructed in The Cancer Genome Atlas (TCGA) cohort and the stability and reliability of the model were validated in the International Cancer Genome Consortium (ICGC) cohort. Assessing the pathogenesis of tumours by ST might provide a better view of the landscape of HCC heterogeneity than traditional methods to facilitate personalized management, and ST cluster-related gene signatures can provide novel antitumour drug targets for HCC.

## Materials and methods

### Patient samples

Tissue samples were obtained from 3 patients who underwent hepatectomy because of HCC in August 2020 from the Tumour Tissue Bank of Tianjin Cancer Hospital. Pathologists confirmed all patients’ diagnoses. Detailed pathologic and clinical data are listed in Additional file 10: Table S1. The use of these tissue samples in this study was approved by the institutional research committee.

### Slide preparation

There were six capture areas (3 tumour areas and 3 peritumour areas) (6.5 × 6.5 mm) of ST slides, each with 4999 capture spots of barcoded primers (10 × Genomics). The diameter of the spots was 100 μm. These spots were arranged in a rectangular shape. Each spot contained millions of oligonucleotides with the following features: a 30-nucleotide poly (dT) sequence for the capture of polyadenylated mRNA molecules; a 12-nucleotide unique molecular identifier (UMI) for the identification of duplicate molecules that arise during the library preparation and sequencing process; a 16-nucleotide spatial barcode, which was shared by all oligonucleotides within each individual gene expression spot; and a partial TruSeq Read 1 sequence for use during the library preparation and sequencing portions of the workflow.

### Tissue preparation, fixation and staining

The 10 × Visium protocol was optimized for frozen tissue. Briefly, tumours were frozen in dry ice immediately after harvesting. Tumours were embedded with optimal cutting temperature (OCT) compound and cryosectioned at 10-μm thick. The sections on the capture areas were placed and incubated them at 37 °C for 1 min and then fixed them in methanol for 10 min at − 20 °C. To stain, sections were incubated in isopropanol (Millipore Sigma) for 6 min, Mayer’s haematoxylin (Dako, Agilent, Santa Clara, CA) for 7 min, bluing buffer (Dako) for 1 min, and eosin (Sigma-Aldrich) diluted 1:5 in Tris-base (0.45 M Tris, 0.5 M acetic acid, pH 6.0) for 1 min. The slides were washed with deionized water after each of the staining steps. After air-drying, the slides were mounted with 85% glycerol and then coverslipped them. Haematoxylin and eosin (H&E)-stained samples were photographed at 40 × magnification using a digital slice scanner (Hamamatsu). The coverslip was removed after imaging by immersing slides in RNase- and DNase-free water.

### Tissue permeabilization, reverse transcription and spatial library preparation

To prepermeabilize the samples, sections were incubated at 37 °C for 24 min with permeabilization enzyme. The wells were washed with saline sodium citrate (SSC) (0.1 ×) (Sigma-Aldrich). SSC was removed, and reverse transcription Master Mix was added to each well. Reverse transcription was conducted according to the ST recommended protocol. After RT, sections were incubated in KOH (0.08 M) for 5 min at room temperature and then incubated in Second Strand Mix for 15 min at 65 °C. After the removal of Second Strand Mix, 100 μl Buffer EB was added, and the sections were incubated in KOH for 10 min at room temperature. The samples were transferred from every single well to a corresponding tube containing Tris–HCl (1 M, pH 7.0). Next, 1 μl of sample was added to the qPCR plate well containing KAPA SYBR FAST qPCR Master Mix (KAPA Biosystems). A qPCR system was used to determine the optimal number of cycles. After that, 65 μl cDNA Amplification Mix was added to the remaining sample. They were incubated according to the recommended protocol.

### Library preparation and RNA sequencing

After the cDNA amplification products were qualified, the sequencing library was constructed with Library Construction Kit (10 × Genomics). First, the cDNA was chemically digested. The cDNA was cut into 200 ~ 300-bp fragments, and the cDNA fragments were segmented and subjected to terminal repair and adaptor ligation. The cDNA fragments were screened. The P7 adaptor was connected and introduced into the sample index by PCR amplification. Finally, the sequence library was obtained. Sequencing was performed on an Illumina HiSeq 3000/4000 with a 150 bp paired-end run by Quick Biology (Pasadena, CA). A data quality check was done on Illumina SAV files. Demultiplexing was performed with the Illumina fastq2 v 2.17 program.

### RNA sequencing analysis

In this study, 10 × Genomics official software Space Ranger was used for data preprocessing, gene expression quantification and point identification. Sequencing data preprocessing included filtering the sequenced sequences, evaluating the quality of sequencing data, and calculating the sequence length distribution. The web-based ST spot detector software Space Ranger was used to identify the spatial barcode markers in Reads1 and UMI markers of different transcripts. Read2 was aligned to the genome using the transcriptome-specific alignment software STAR, and sequences with unique alignment positions were selected for subsequent analysis. The gene spot matrix was generated by using Visium spatial barcodes, and then point clustering and gene expression analysis were performed. Seurat software was used to analyse and cluster the samples. Low-quality data points were filtered out. Principal component analysis (PCA), including the t-distributed stochastic neighbour embedding (t-SNE) and uniform manifold approximation and projection (UMAP) algorithms, was used to reduce the dimensionality of the data and visualize the data.

### Data quality control and normalization with Space Ranger

Sequencing data Read1 contained barcode and UMI markers that distinguished transcripts. The 10 × Genomics official analysis software Space Ranger was used to statistically analyse the UMI-related quality control information of each sample. To understand the biological significance of expression differences in different locations, it is necessary to classify the points according to the expression level. The points with similar expression levels may come from the same type of sample. Space Ranger software can be used to preliminarily classify different locations on the genome. First, the expression of all points was normalized to compare the expression. Then, t-SNE dimension reduction analysis based on PCA was carried out, and the t-SNE results were clustered.

### Identification of tumour cluster-specific marker genes (DEGs)

Gene sets with FDR-adjusted *P*-values below 0.05 were considered significantly enriched in the related clusters, and these genes were identified as DEGs. Kyoto Encyclopedia of Genes and Genomes (KEGG) and gene ontology (GO) analyses were used to analyse the signalling pathways involved. Marker genes were identified based on the comprehensive analysis of the database and gene rank of the log fold change (FC) value of DEGs in the clusters. Pearson correlation analysis was used to reveal the relationship between cluster-specific genes and marker genes.

### Clinical significance of tumour cluster-specific marker genes in HCC

The prognostic value of tumour cluster-specific marker genes was evaluated in the KM Plotter database. The genes were submitted to the website. Approximately 155 HCC patients were split into two groups according to the median value of marker gene expression from the gene chip. These survival of the two groups of patients was compared with a Kaplan–Meier (KM) survival plot.

### Data collection (TCGA-LIHC cohort and ICGC (LIRI-JP) cohort)

RNA sequencing data and the corresponding clinical information of 376 patients with liver cancer up to July 13, 2021 were downloaded from the TCGA website (https://portal.gdc.cancer.gov/repository). The RNA sequencing data and clinical information of another 260 patients were obtained from the ICGC website (https://dcc.icgc.org/releases/current/Projects/LIRI-JP). The data from the TCGA and ICGC databases are public. Thus, the present study was exempt from the requirement for the approval of local ethics committees. The current research follows TCGA and ICGC data access policies and publication guidelines.

### Construction and validation of a prognostic spatial cluster-related gene signature

Univariate Cox analysis of overall survival (OS) was utilized to screen tumour cluster-specific marker genes with prognostic value. P values were adjusted by Benjamini & Hochberg (BH) correction. Least absolute shrinkage and selection operator (LASSO)-penalized Cox regression analysis was performed to construct a prognostic model to minimize the risk of overfitting [6]. The LASSO algorithm was used for variable selection and shrinkage with the “glmnet” R package. The normalized expression matrix of candidate prognostic DEGs was the independent variable in regression, and the dependent variables were the OS and risk status of patients in the TCGA cohort. The risk scores of the patients were calculated according to the normalized expression level of each gene and its corresponding regression coefficients. The formula was established as follows: score = esum (each gene’s expression × corresponding coefficient). The patients were stratified into high-risk and low-risk groups based on the median value of the risk score. Based on the expression of genes in the signature, PCA and t-SNE analysis were performed with the “Rtsne” and “ggplot2” R packages to explore the distribution of different groups. Survival analysis was implemented to analyse the OS of high- and low-risk groups using the “survminer” R package. The “survival ROC” R package was used to conduct time‐dependent receiver operating characteristic (ROC) curve analyses to evaluate the predictive power of the gene signature. The infiltration scores of 16 immune cells and the activities of 13 immune-related pathways between the high- and low-risk groups were calculated by single-sample gene set enrichment analysis (ssGSEA) with the “GSVA” R package.

Immunohistochemical staining and scoring (Additional file 1: Materials and methods).

### Statistical analysis

The chi-squared test was used to compare the different proportions. Student’s t test was used to compare the image scores. The ssGSEA scores of immune cells or immune pathways between the high- and low-risk groups were compared by the Mann–Whitney test, and the P value was adjusted by the Bejamini-Hochberg (BH) method. KM analysis was employed to compare the differences in OS among different groups. Univariate and multivariate Cox analyses were performed to screen the independent predictors for OS. The correlation of the prognostic model risk score or prognostic gene expression level with the stromal score, immune score and drug sensitivity was tested by Spearman or Pearson correlation analysis. R software (Version 4.0.5) with the packages Venn, igraph, ggplot2, pheatmap, ggpubr, corrplot and survminer was used to create plots. For all statistical results, a two-tailed P value less than 0.05 indicated statistical significance.

## Results

Cluster-specific marker genes (DEGs) were analysed from two perspectives; therefore, the results are presented in two parts.

### The establishment of marker gene profiles for the prediction of satellite nodules

**Fig. 1:**
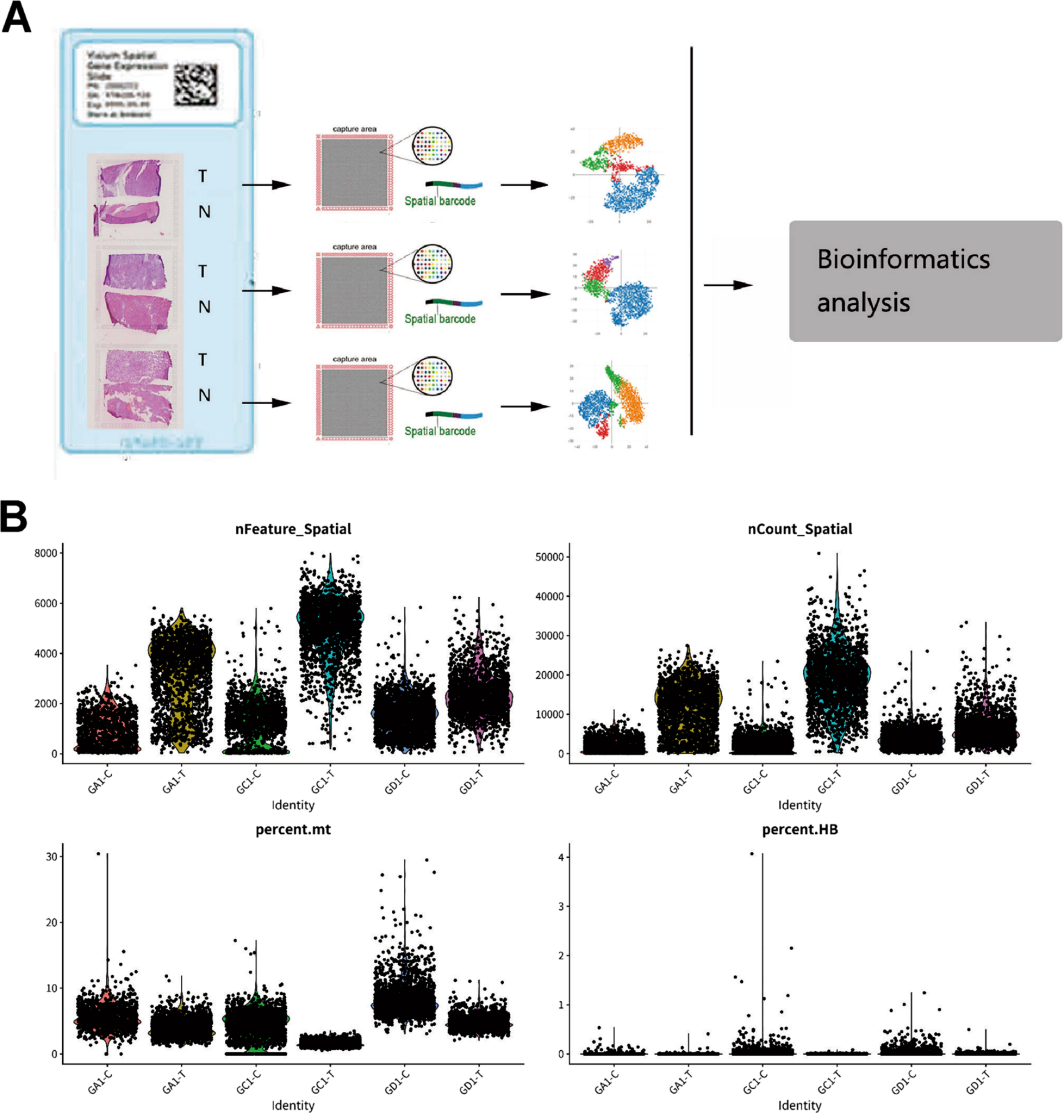
Study design for ST in HCC. **A** Workflow for HCC ST. HCC specimens of three patients were dissected to separate bulk tumours from peritumour tissue. Three pairs of tumour and peritumour tissues were analysed. **B** The distribution of all expressed gene numbers, distribution of all expressed genes, distribution of mitochondrial genes and distribution of haemoglobin gene expression in three pairs of samples

The flow chart of first part is shown in Fig. 1. To spatially analyse the gene expression of each HCC, 3 individual patient samples (one with satellite nodules and two without satellite nodules) were analysed using the ST methodology. For each specimen, the bulk tumour were separated from the adjacent peritumoural tissue. ST was performed to detect the spatial gene expression of 6 tissue sections. Figure 1 shows the gene number distribution, expression distribution, mitochondrial genes, and haemoglobin gene expression ratio of all the spots. This figure also indicates the spatial and expression distribution of genes in 6 sections. Overall, 10,820 tissue regions were analysed within the 6 samples.

### Spatial transcriptome heterogeneity in HCC

**Fig. 2:**
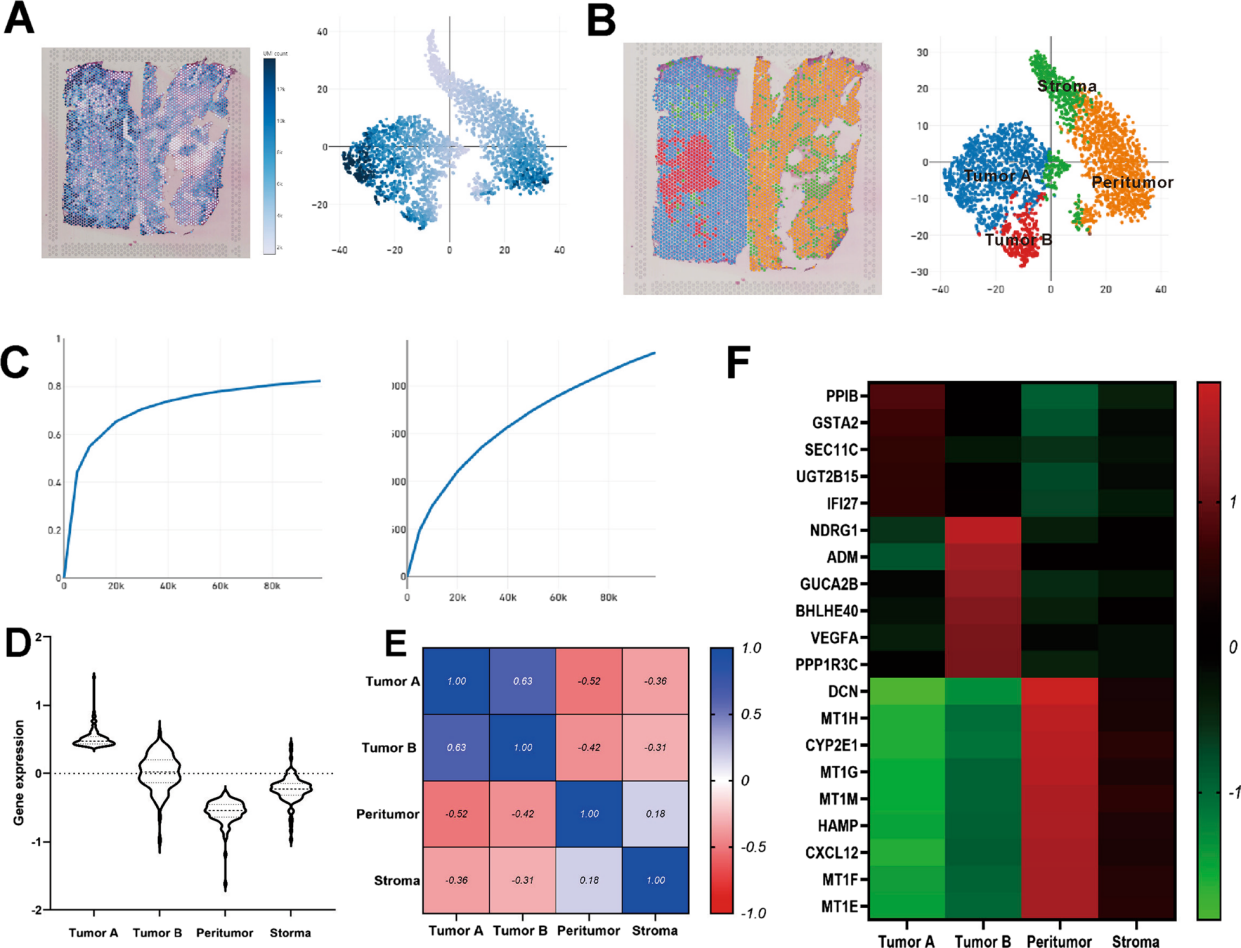
Spatial gene expression heterogeneity within the case 1 tissue sample. **A** Tissue plot with spots coloured by UMI count and t-SNE projection of spots coloured by UMI counts. **B** Tissue plot with spots coloured by clustering and t-SNE projection of spots coloured by clustering. **C** Sequencing saturation and median genes per spot. **D** Violin plots displaying the expression of the top 100 DEGs in the 4 main clusters. **E** Spearman correlation between tumour area versus peritumour area. **F** Heatmap showing the expression levels of specific markers in 4 clusters

One pair of tissue sections (tumour and peritumoural tissue with satellite nodules) was initially analysed. To analyse the different components within the cell in HCC, PCA was used to analyse the DEGs among all cells. According to the results of t-SNE and UMAP, all spots of 2 sections were grouped into 4 main clusters (Fig. 2A–C). These clusters showed spatial morphological characteristics that closely reflected those of histologically identifiable structures, including tumours, peritumour tissue and stroma. Each cluster had unique differentially expressed genes, indicating intratumour and intertumour heterogeneity in HCC (Fig. 2D–F). Analysis of these 4 clusters showed that gene expression was similar between peritumour tissues but significantly different between peritumour and tumour tissues.

A weaker correlation was observed between tumour samples than between peritumoural tissues. This result suggested that intertumour heterogeneity was more pronounced (Fig. 2E). Thereafter, differential gene expression analysis were performed to identify cluster-specific marker genes and then defined the identity of each cluster (Fig. 2F). The gene expression profiles represent the HCC expression phenotype while taking tissue origin or the functional respective tissue components into account.

### ST analysis uncovered changes that could not be detected by single-cell transcriptomics analysis

**Fig. 3:**
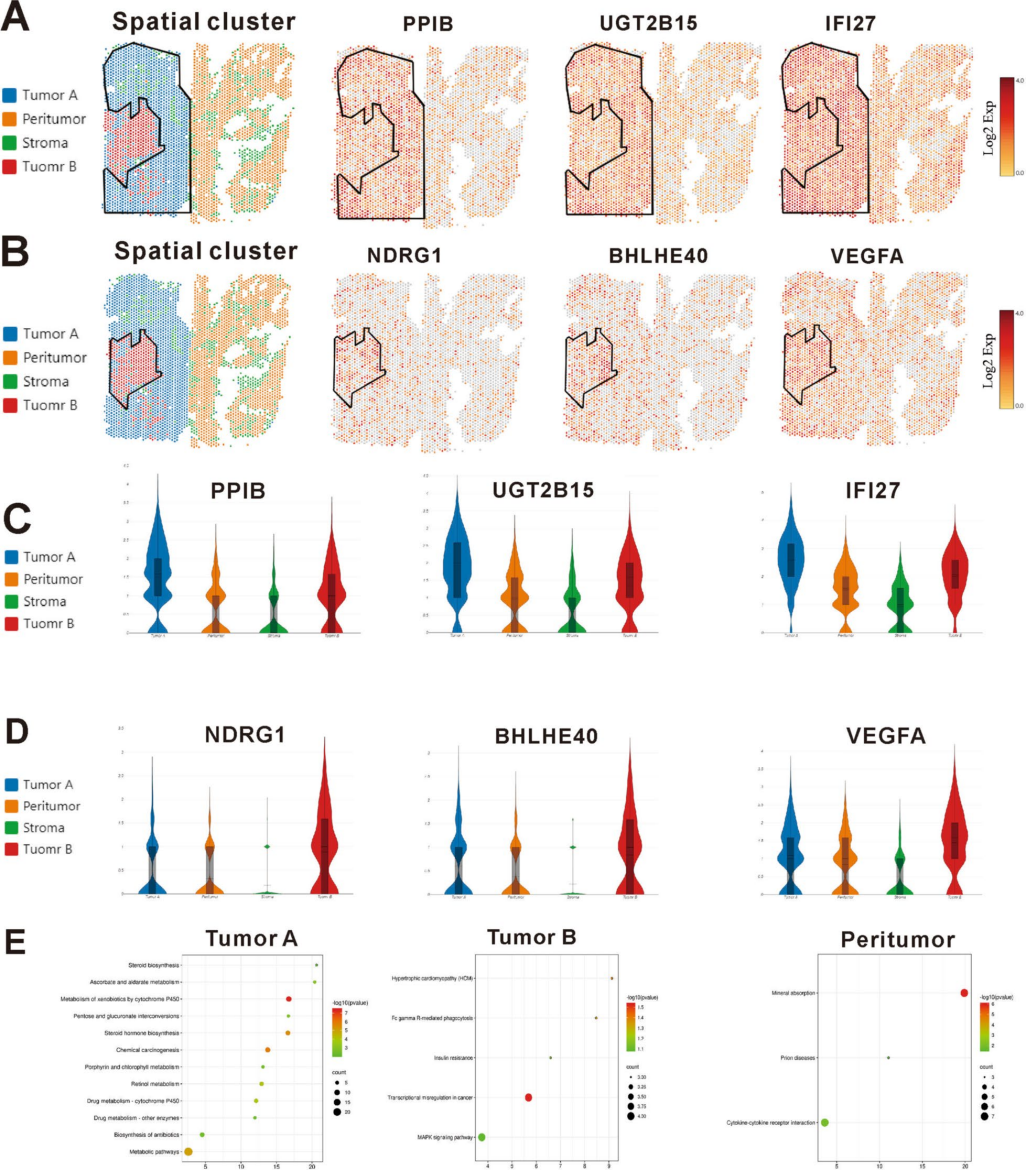
Spatial gene expression comparison of tumour cluster A and cluster B. **A** Gene expression in tumour cluster A. **B** Gene expression in tumour cluster B. **C** and **D** Violin plots displaying the expression of representative marker genes identified in tumour and peritumour clusters. **E** Pathways enriched in tumour and peritumour clusters

Interestingly, there were two tumour clusters in case 1, which had satellite nodules, while there was only one tumour cluster in the other two cases, which did not have satellite nodules. To understand the possible molecular mechanism of intrahepatic metastases, we sought to investigate functional differences in gene expression between these two tumour clusters in more detail. In tumour cluster A, *PPIB, UGT2B15 and IFI27* were among the most highly expressed genes (Fig. 3A and C), and their functions were related to cell survival and apoptosis. In tumour cluster B (Fig. 3B and D), *NDRG1, BHLHE40* and *VEGFA*, all of which have functions related to metastasis and invasion, were among the most highly expressed genes. In addition to our gene expression analysis, pathways in two tumour clusters were investigated (Fig. 3E). The pathways activated in tumour cluster A are mainly linked to altered cellular metabolism (metabolic pathways, drug metabolism, ascorbate and aldarate metabolism and steroid biosynthesis). Metabolic alteration is a hallmark of cancer [7] and a clear characteristic of HCC [8]. A large number of clinical parameters are used to evaluate liver function, reflecting changes in both enzyme activity and metabolites. On the other hand, we found that GO categories linked to transcriptional misregulation in cancer and the MAPK signalling pathway were enriched in tumour cluster B of case 1, possibly reflecting increased cell proliferation and malignancy.

### Spatial expression patterns common to HCC samples

Next, gene expression analyses for another two cases were performed. The resulting gene expression profiles of tumour and nonneoplastic tissues were similar (Additional file 2: Fig. S1). The gene expression within each region (tumour, peritumour and stroma) obtained from the previous results was used to determine region-specific markers. The expression of some specific genes was observed to be higher in the tumour than in the nonneoplastic tissues in each case. Specifically, in the tumour region, we observed enrichment of *PPIB* and *UGT2B15* and *IFI27, NDRG1, BHLHE40* and *VEGFA* (Additional file 3: Fig. S2).

We then investigated more functional differences in pathways between the tumour and the peritumour region (Additional file 4: Fig. S3). Consistent with the pathway analysis in case 1, enrichment analysis indicated pathways enriched in the tumour (for example, genes involved in chemical carcinogenesis and metabolic pathways) versus the peritumour tissue.

In summary, the gene profiles obtained from the ST analyses may reflect the different statuses of HCC and can reveal intertumour heterogeneity between patients at the gene expression level.

### The effect of marker gene expression in tumour clusters on HCC patient prognosis

**Fig. 4:**
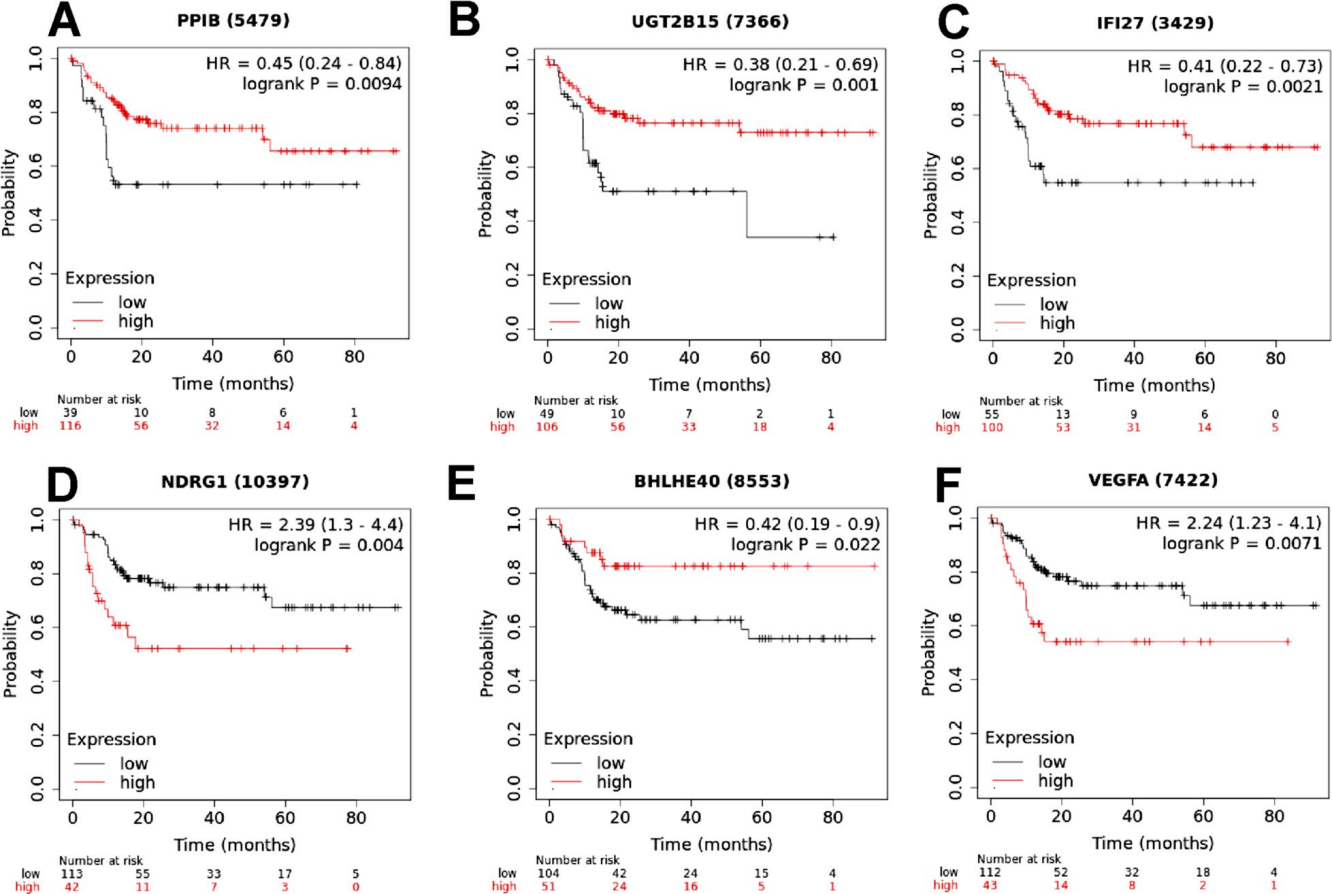
KM survival plot of the top 6 marker genes of tumour clusters. **A** KM survival plot of *PPIB*. **B** KM survival plot of *UGT2B15*. **C** KM survival plot of *IFI27*. **D** KM survival plot of *NDRG1*. **E** KM survival plot of *BHLHE40.* **F** KM survival plot of *VEGFA*

To evaluate the clinical significance of marker genes of tumour clusters, the KM plotter website was applied. According to analysis of 155 HCC samples in the KM Plotter database, high expression levels of *PPIB, UGT2B15, IFI27, NDRG1, BHLHE40* and *VEGFA* were associated with a poor prognosis in HCC (Fig. 4).

### Construction of the prognostic gene signature

The flow chart of the second part is shown in Additional file 5: Fig. S4 total of 361 HCC patients from the TCGA-LIHC cohort and 260 HCC patients from the ICGC (LIRI-JP) cohort were finally enrolled. The detailed clinical characteristics of these patients are summarized in Table 1.

**Table 1:** Clinical characteristics of the HCC patients used in this study

Characteristics	TCGA-LIHC cohort	ICGC-LIRP-JI cohort
No. of patients	371	260
Age (median, range)	61 (16–90)	69 (31–89)
Gender		
Female	120 (32.53%)	68 (26.15%)
Male	251 (67.65%)	192 (73.85%)
Grade		
G1	55	NA
G2	178	NA
G3	120	NA
G4	13	NA
Unknown	5	NA
Stage		
Stage I	174	40
Stage II	85	117
Stage III	84	80
Stage IV	4	23
Unknown	24	NA
Survival time		
Alive	239	214
Deceased	132	46

### Identification of prognostic spatial cluster-specific marker genes (DEGs) and construction of a prognostic model in the TCGA cohort

**Fig. 5:**
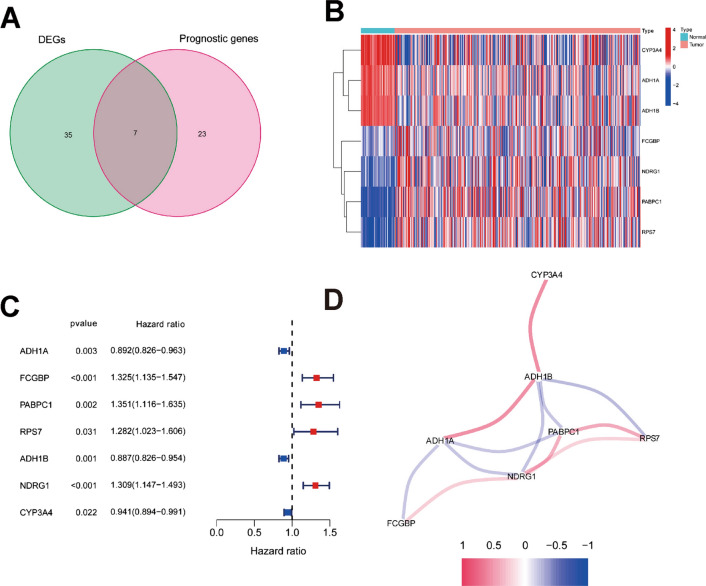
Identification of candidate spatial tumour cluster-specific genes in the TCGA cohort. **A** Venn diagram to identify cluster-specific marker genes (DEGs) correlated with OS between HCC tissues and adjacent normal tissues. **B** The expression of 7 spatial cluster-specific marker genes between HCC tissues and adjacent normal tissues. **C** Forest plots showing the results of the univariate Cox regression analysis between gene expression and OS. **D** The correlation network of candidate genes

**Fig. 6:**
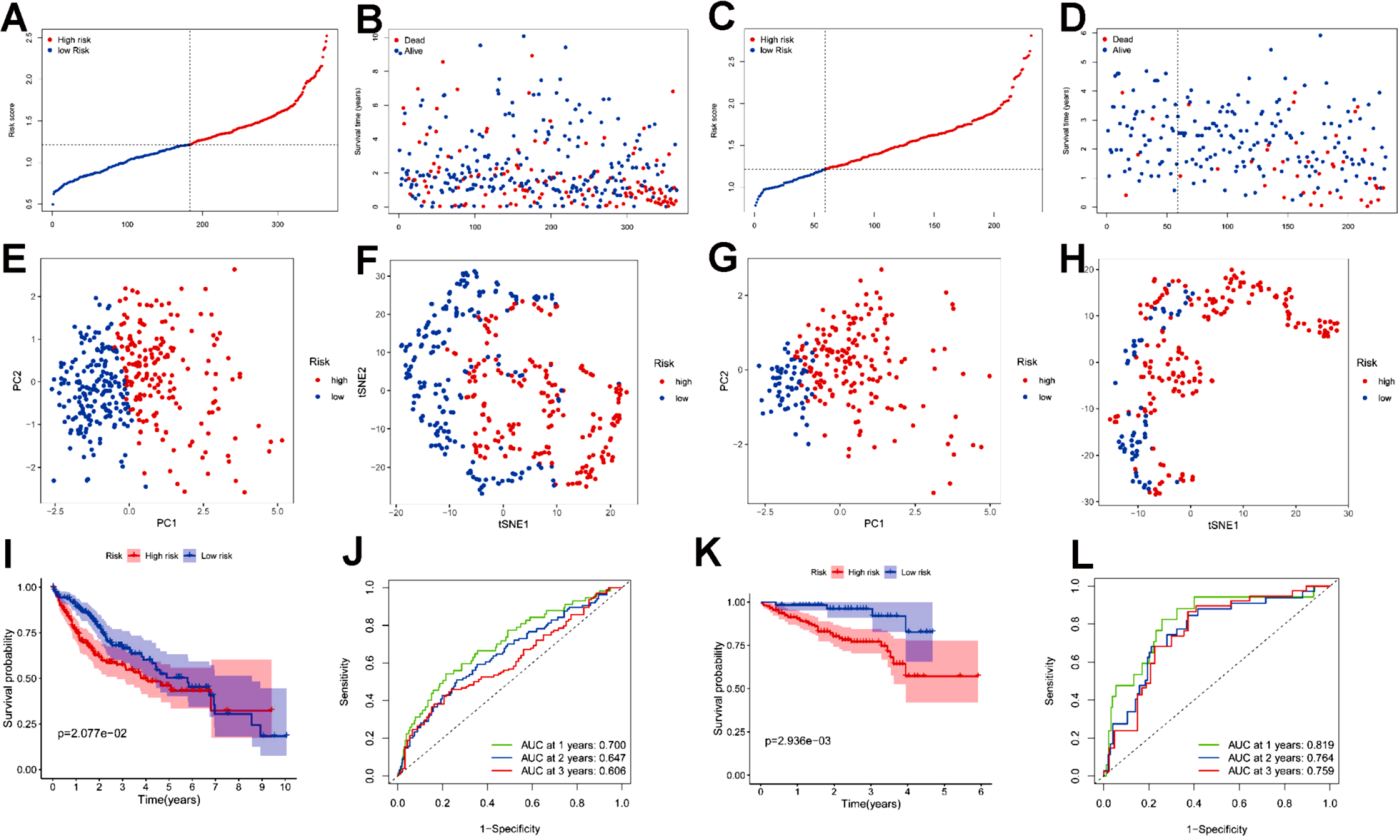
Prognostic analysis of the 6-gene signature model in the TCGA cohort and ICGC cohort. TCGA cohort (**A**, **B**, **E**, **F**, **I**, **J**), ICGC cohort (**C**, **D**, **G**, **H**, **K**, **L**). **A**, **C** The median value and distribution of the risk scores. **B**, **D** The distribution of OS status. **E**, **G** PCA plot. **F**, **H** t-SNE analysis. **I**, **K** KM curves for OS of patients in the high- and low-risk groups. **J**, **L** AUC time-dependent ROC curves for OS

Spatial cluster-specific marker genes (DEGs) were differentially expressed in tumour tissues and adjacent nontumour tissues, and 7 of them were correlated with OS in the univariate Cox regression analysis (Fig. 5A). The expression profiles of the above 7 genes were analysed by LASSO-Cox regression analysis, and a prognostic model was established. *RPS7* was excluded from this analysis because its LASSO coefficient was unavailable. A 6-gene signature (*ADH1A, ADH1B, CYP3A4, FCGBP, PABPC1, NDRG1*) was identified based on the optimal value of λ (Additional file 6: Fig. S5). The risk score was calculated as follows: score = -0.0354 × expression level of *ADH1A*—0.0035 × expression level of *ADH1B* − 0.0100 × expression level of *CYP3A4* + 0.113 × expression level of *FCGBP* + 0.119 × expression level of *PABPC1* + 0.126 × expression level of *NDRG1*. Figure 5B shows that the expression of these 6 genes was significantly different in HCC tissues and adjacent normal tissues. The risk ratio of *NDRG1*, an important marker from tumour cluster B, was 1.309 (95% CI = 1.147–1.493, *P* < 0.001, Fig. 5C). Figure 5D shows the relationship of those 6 genes. The results showed that these 6 spatial cluster-specific marker genes can be used as prognostic indicators. Based on the median cut-off value, we divided the patients into a high-risk group (n = 182) or a low-risk group (n = 183) (Fig. 6A). We found that high risk was correlated with a higher tumour grade (Table 2). Patients with low risk had longer survival times than those with high risk according to the scatter plot (Fig. 6B). The patients were distributed in the two subgroups according to whether they were in the high- or low-risk group through PCA and t-SNE analysis (Fig. 6E–F). On the other hand, the survival analysis showed that the patients with low risk had a better 5-year OS than those with high risk (Fig. 6I, P < 0.05). Time-dependent ROC curves were generated to display the sensitivity and specificity of the survival prognostic model, and the area under the curve (AUC) reached 0.700 at 1 year, 0.647 at 2 years, and 0.606 at 3 years (Fig. 6J).

**Table 2:** Characteristics of the patients in different risk groups

Characteristics	TCGA-LIHC cohort			ICGC-LIRP-JI cohort		
	High risk	Low risk	P value	High risk	Low risk	P value
Age						
≤ 65	118 (69.01%)	101 (60.12%)	0.1102	67 (38.95%)	22 (37.29%)	0.9428
> 65	53 (30.99%)	67 (39.88%)		105 (61.05%)	37 (62.71%)	
Gender						
Female	54 (31.58%)	54 (32.14%)	1	49 (28.49%)	12 (20.34%)	0.2918
Male	117 (68.42%)	114 (67.86%)		123 (71.51%)	47 (79.66%)	
Grade						
G1 + 2	87 (50.88%)	125 (74.4%)	< 0.001	NA	NA	NA
G3 + 4	84 (49.12%)	43 (25.6%)		NA	NA	NA
Stage						
Stage I–II	121 (70.76%)	134 (79.76%)	0.0729	96 (55.81%)	45 (76.27%)	0.0086
Stage III–IV	50 (29.24%)	34 (20.24%)		76 (44.19%)	14 (23.73%)	

To explore the relationship between each prognostic gene and prognosis, survival analysis was performed. The results indicated that low expression of *ADH1A, CYP3A4* and *ADH1B* was significantly correlated with poor OS (Additional file 7: Fig. S6A–C, *P* < 0.01), and high expression of *FCGBP, PABPC1* and *NDRG1* was significantly correlated with poor OS (Additional file 7: Fig. S6D-E and Fig. 4D, P < 0.05). The expression levels of *FCGBP, PABPC1* and *NDRG1* were higher and the expression levels of *ADH1A, CYP3A4* and *ADH1B* were lower in tumour tissues than in adjacent nontumour tissues (Additional file 8: Fig. S7). Immunohistochemical studies have also been performed to validate the clinical significance of these marker genes in HCC models (34 pairs of HCC and adjacent nontumour tissues) (Additional file 9: Fig. S8) and the statistical analysis of image scores showed consistent results (Additional file 11: Table S2).

### Validation of the 6-gene signature in the ICGC Cohort

To further validate the stability of the model based on the TCGA cohort, we performed the same analysis with the ICGC cohort. Referring to the median value obtained from the TCGA cohort, patients from the ICGC cohort were also divided into two groups (high or low risk). Consistent with the results from the TCGA cohort, PCA and t-SNE analyses confirmed that patients were separated in two groups (Fig. 6G, H). Patients in the low-risk group were less likely to die earlier (Fig. 6D) and had a longer survival time than those in the high-risk group (Fig. 6K). Moreover, the AUC of the 6-gene signature was 0.819 at 1 year, 0.764 at 2 years, and 0.759 at 3 years (Fig. 6L).

### Independent prognostic value of the 6 gene signature

**Fig. 7:**
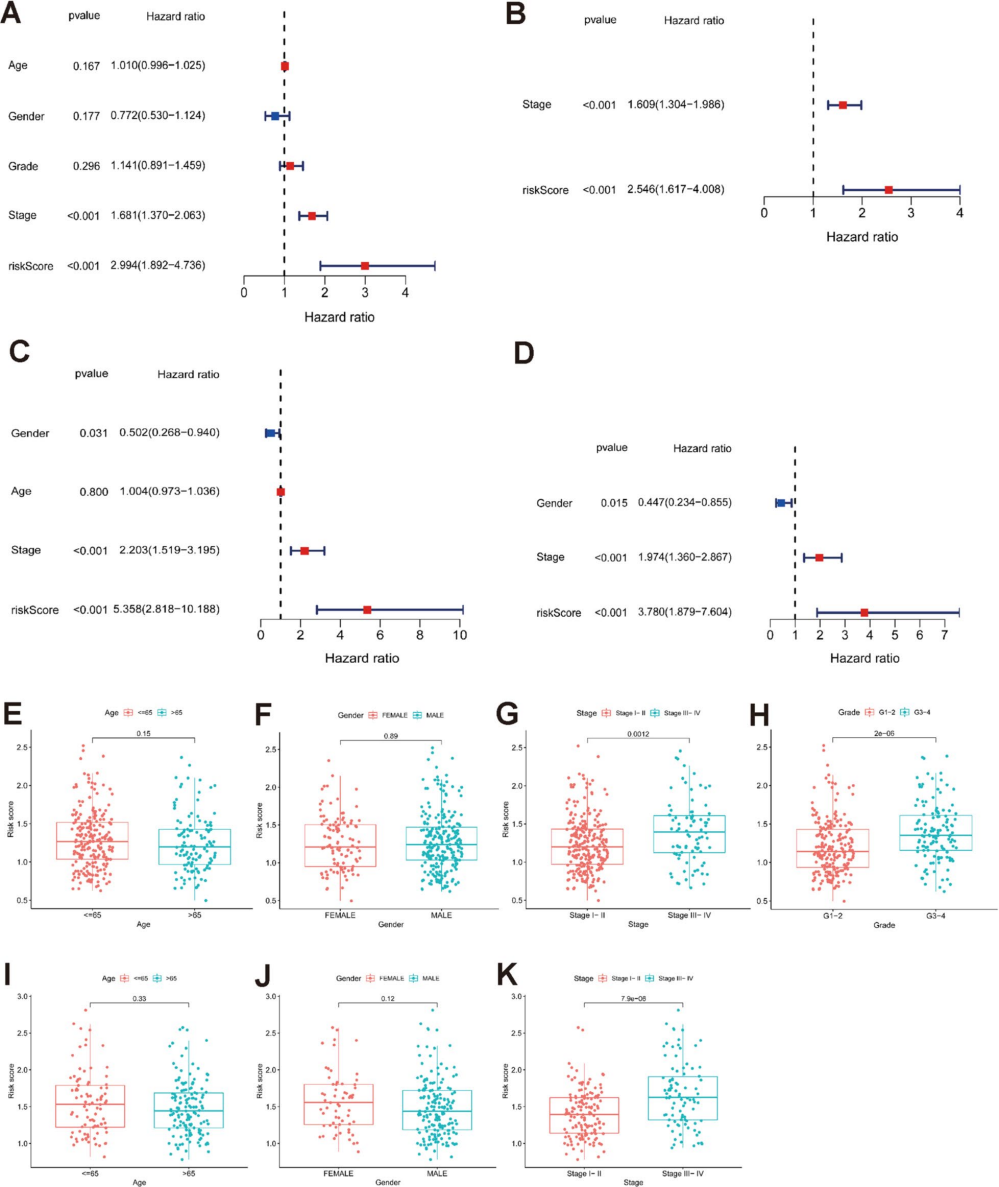
The risk score in different groups divided by clinical characteristics and results of the univariate and multivariate Cox regression analyses. TCGA cohort (**A**–**D**, **H**, **J**), ICGC cohort (**E**–**F**, **I**, **K**). **A**, **E** Age. **B**, **F** Gender. **C** Tumour stage. **D**, **G** Tumour grade. **H**, **I** OS-related factors were screened by univariate Cox regression analyses. **J**, **K** OS-related factors were screened by multivariate Cox regression analysis

To validate whether the risk score was an independent prognostic factor for OS, we carried out univariate and multivariate Cox analyses. In both the TCGA and ICGC cohorts, the risk scores were significantly correlated with OS according to univariate Cox analysis (TCGA cohort: HR = 2.994, 95% CI = 1.892–4.736, P < 0.001; ICGC cohort: HR = 5.358, 95% CI = 2.818–10.188, P < 0.001) (Fig. 7A, C). Even after correcting for other factors, the risk score was still an independent predictor for OS based on multivariate Cox analysis (TCGA cohort: HR = 2.546, 95% CI = 1.617–4.008, P < 0.001; ICGC cohort: HR = 3.780, 95% CI = 1.879–7.604, P = 0.001) (Fig. 7B, D).

### Functional analyses with the TCGA and ICGC cohorts

The association of the risk score with the clinical characteristics of HCC patients was analysed. The results showed that the risk score was significantly higher in tumour stage III-IV (P < 0.001) or grade 3–4 (P < 0.001) than in tumour stage I-II (Fig. 7G, H) or tumour grade 1–2. The same analysis of the ICGC dataset confirmed that the risk score was definitely higher in tumour stages III-IV than in tumour stages I-II (P < 0.001) (there were no data about the grade of HCC in the ICGC dataset) (Fig. 7K).

To detect whether the risk score was associated with immune components, we analysed the relationship of the risk score and immune infiltration.

**Fig. 8:**
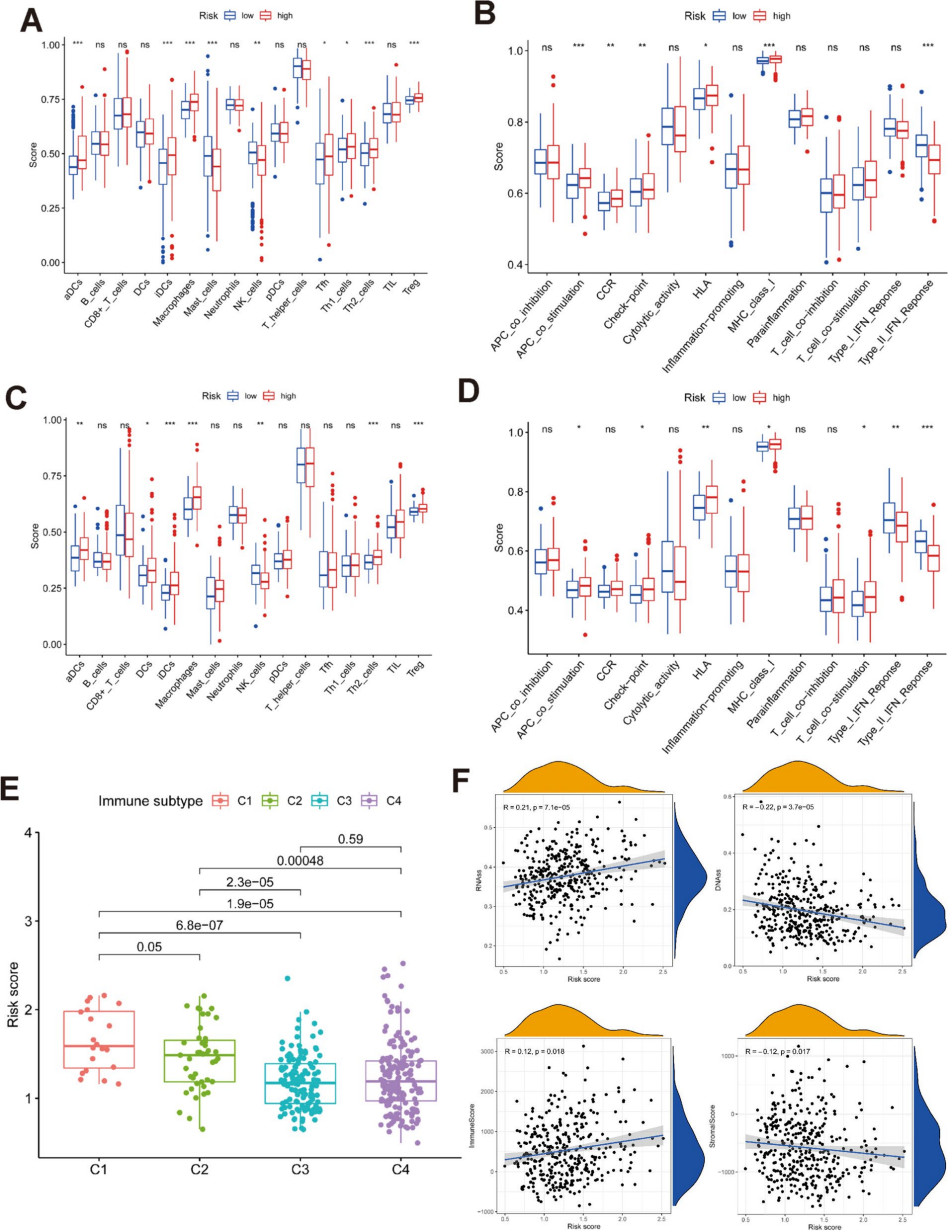
Immune status between different risk groups and the association between the risk score and tumour microenvironment scores. TCGA cohort (**A**, **C**), ICGC cohort (**B**, **D**). **A**, **B** The scores of 16 immune cells and **C**, **D** 13 immune-related functions are shown in boxplots. **E** Comparison of the risk score in different immune infiltration subtypes. **D** The relationship between risk score and RNAss, DNAss, stromal score and immune score. P values are shown as ns, not significant; *P < 0.05; **P < 0.01; ***P < 0.001

Factors such as dendritic cells (DCs), interstitial dendritic cells (iDCs), antigen-presenting cell (APC) costimulation, human leukocyte antigen (HLA), and major histocompatibility complex (MHC) class 1, which are important for the antigen presentation process, were significantly induced in the high-risk group in the TCGA cohort (all adjusted P < 0.05, Fig. 8A, B). In addition, the scores of macrophages or Treg cells were higher in the high-risk group, while the scores of the type II IFN response and mast cells showed the opposite trend (adjusted P < 0.05, Fig. 8A, B). Comparison between high- and low-risk groups of the ICGC cohort showed a similar result as that in the TCGA cohort analysis (adjusted P < 0.05, Fig. 8C, D).

Six types of immune infiltrates were identified in human tumours, namely, C1 (wound healing), C2 (INF-g dominant), C3 (inflammatory), C4 (lymphocyte depleted), C5 (immunologically quiet) and C6 (TGF-γ dominant) [9]. The C5 and C6 immune subtypes were not included in the study because no patient sample belonged to the C5 immune subtype and C6 immune subtype in HCC. The relationship were analysed between immune infiltration and the risk score. We observed that a high risk score was significantly associated with C2, while a low risk score was significantly associated with C3 (Fig. 8E). Tumour stemness was also assessed according to the RNA stemness score (RNAss) based on mRNA expression and the DNA stemness score (DNAss) based on the DNA methylation pattern [10]. The tumour immune microenvironment was assessed with the stromal score and immune score. Correlation analysis was also performed to explore the relationship between tumour stem cells and the immune environment. Based on the results, we can see that the risk score was positively significantly correlated with DNAss and immune score but significantly negatively associated with RNAss and stromal score (P < 0.05) (Fig. 8F).

**Fig. 9:**
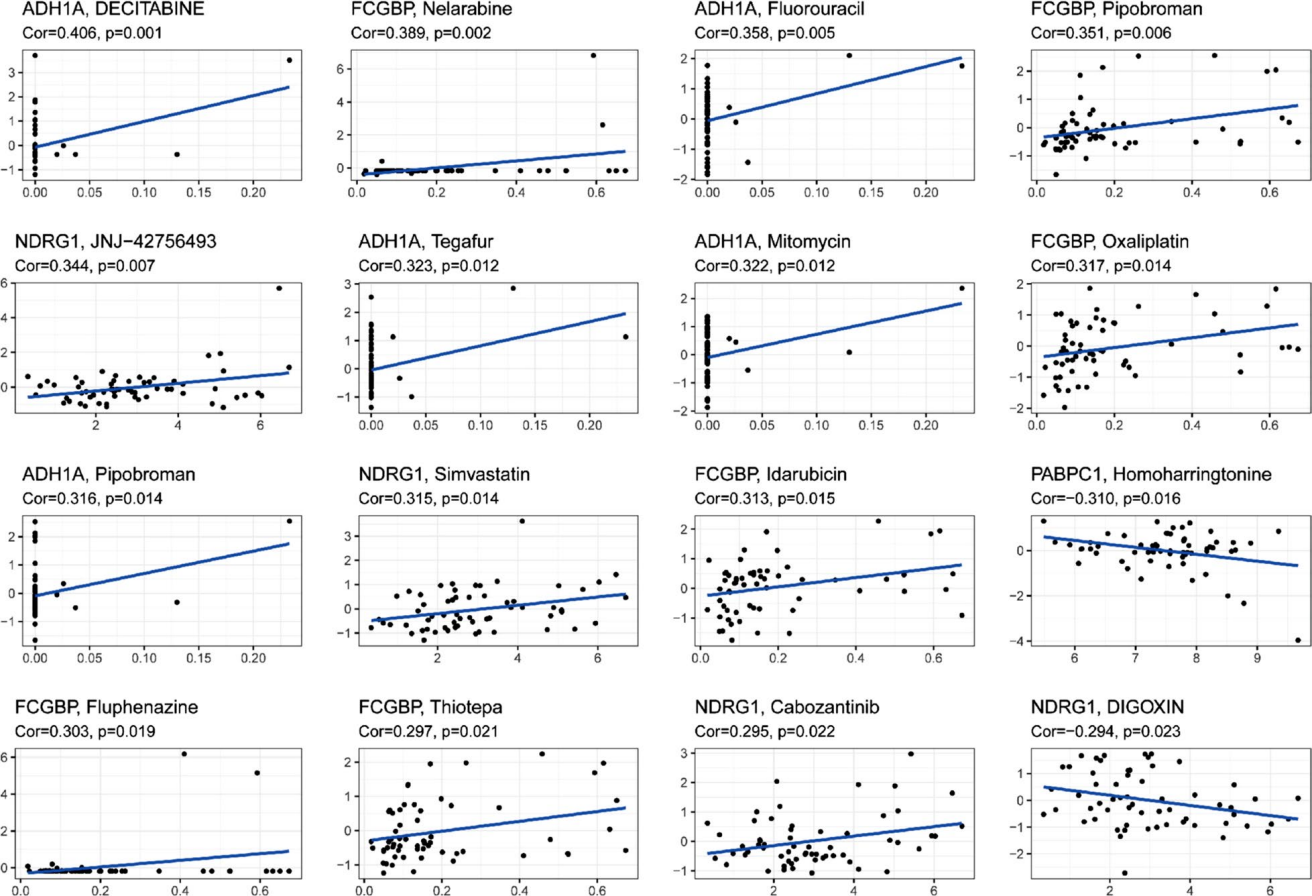
Scatter plot of the relationship between the expression of prognostic genes and drug sensitivity

The relationship between the expression of prognostic genes and drug sensitivity was also examined. The results showed that all prognostic genes were correlated with the sensitivity to some chemotherapies (P < 0.01) (Fig. 9). For example, *NDRG1* expression was increased with JNJ-42756493, simvastatin and cabozantinib.

## Discussion

HCC treatment outcomes are still not promising because of the high recurrence rate even after complete surgical excision. The presence of satellite lesions missed by imaging because of their small size may be one of the key factors leading to high recurrence. The purpose of the present study was to perform a full analysis of the spatial transcriptome of HCC with satellite nodules. Tissue-wide gene expression heterogeneity was investigated using ST technology, which quantifies transcriptome arrays of whole tissue sections.

Recently, a study including multiple histological types of cancer suggested that adjacent tumour tissue may be an intermediate state between normal and tumour tissue [11]. However, no evaluation of tumour and peritumour tissue with spatial resolution has been conducted until now. Therefore, we identified differences between the tumour and peritumour specimens. The results showed that the gene expression and pathway enrichment were different in tumour and peritumour clusters. For example, known HCC-related genes (*NDRG1* [12, 13] and *VEGFA* [14]) are among the most highly expressed genes in the “tumour” clusters. These highly expressed gene profiles may become gene markers of HCC, suggesting poor prognosis. The present study also offers a new perspective into gene expression differences between tumours with and without satellite nodules, prompting key questions with important implications for the metastasis of HCC. The results revealed two tumour clusters in the case with satellite nodules. We identified marker genes corresponding to the different clusters of tumour cells. The gene expression profiles obtained from the ST analysis may be used to predict additional regions with satellite nodules. *PPIB, UGT2B15* and *IFI27*, which were found in tumour cluster A, were mainly associated with cell survival and apoptosis. *PPIB* (cyclophilin B, CypB) is a member of the PPIase family. It has been reported to play an important role in protein folding. Recent studies have shown that HCC cell survival can be stimulated by *PPIB* through a positive feedback loop with hypoxia-inducible factor-1a (HIF-1a) [15]. *PPIB* is associated with malignant progression, and gene regulation has been noted by some researchers [16]. Overexpression of miR-206 promotes apoptosis and inhibits the metastasis of HCC cells by targeting *PPIB* [17]. *UGT2B15* is a functional member of the *UGT2B* subfamily. The expression of *UGT2B15* is mainly observed in liver, prostate and breast cancer. It has been found to contribute to glucuronidation of androgenic steroids [18–20]. The role of UGT2B15 in inducing tumour progression and drug resistance has been reported in some studies [20]. Bioinformatic analysis suggests that *UGT2B15* activates the Hippo‑YAP signalling pathway, leading to the pathogenesis of gastric cancer [20]. Interferon alpha-inducible protein 27 (*IFI27*) consists of 122 amino acids. It belongs to a hydrophobic mitochondrial protein family [21]. *IFI27* maintains a low level of expression in multiple mammalian cells and is involved in a wide range of biological processes, such as apoptosis and innate immunity [22, 23]. The development of tumours can be affected by *IFI27* downregulation in many cancers. TRAIL-induced apoptosis in animal and cellular models of HCC and gastric cancer can be induced when *IFI27* is downregulated. This result suggests that *IFI27* may play a critical important role in tumour development [24]. IFI27 downregulation results in a decrease in the formation of the cyclin A/CDK1 complex, inducing epithelial cell arrest in S phase, and cell proliferation is therefore inhibited [25]. Hong W reported that *IFI27* upregulation promotes cell proliferation and invasion and reduces apoptosis [26]. *NDRG1, BHLHE40* and *VEGFA,* which were found in tumour cluster B, mainly affect tumour proliferation, metastasis and invasion. N-Myc downstream-regulated gene 1 (*NDRG1*) is a crucial cytosolic ubiquitously expressed protein. *NDRG1* is an important molecule in controlling HCC metastasis and is thus suggested as a novel biomarker for predicting HCC recurrence after liver transplantation [13]. *NDRG1* is a significant marker for metastasis, recurrence and poor prognosis in HCC [13]. Yan et al. [27] found that *NDRG1* expression is generally upregulated in HCC tissues compared with normal samples, particularly in recurrent and metastatic HCC. *BHLHE40* (also known as *DEC1/BHLHB2/SHARP2/STRA13*) belongs to the basic helix-loop-helix (bHLH) protein family, which is a large superfamily of transcriptional regulators expressed in many organisms. High expression of *BHLHE40* is significantly correlated with the activation of a hypoxia-response pathway, elevated metastatic potential, and a poor prognosis in many tumours, such as HCC, pancreatic cancer, and invasive breast cancer [28–30]. *BHLHE40* is activated under hypoxic conditions by HIF-1α in HCC, stimulating tumour progression [31]. Vascular endothelial growth factor (VEGF) is an essential angiogenic growth factor in physiological and pathological states. High expression of *VEGFA* has been detected in a large number of solid tumours, including HCC [32, 33]. It is involved in the regulation of the metastasis of many solid tumours and their neovasculature [14, 34]. In HCC, VEGF is an extremely important angiogenic factor. *VEGFA* secreted by tumour cells promotes an epithelial-to-mesenchymal transition phenotype, consequently inducing tumour invasion [35]. In our study, spatial gene expression was measured in HCC tissues for the first time.

Spatially mapping gene expression in one case uncovered a new landscape [36], and another two cases were used to validate the results. The results showed that these marker genes in tumour clusters were also highly expressed in the tumour area. We also detected the clinical significance of marker genes through the database and found that these genes can be used to predict prognosis and survival in patients with HCC. In the future, relevant panels can be detected, and molecules in different regions can help improve the accuracy of clinical predictions, of which cases of HCC may be at a higher risk of metastasis. High expression of the markers in both clusters may suggest that there are satellite nodules in tumours.

To further explore the role of spatial cluster-specific marker genes (DEGs), we screened the DEGs in the TCGA and ICGC cohorts. The expression of tumour cluster marker genes was significantly different between tumour and adjacent nontumour tissues, and many of them were associated with OS according to univariate Cox regression analysis. These results suggested that we could construct a prognostic model using cluster-specific marker genes. Patients were divided into high- and low-risk groups according to the median risk score. The results showed that high risk was significantly correlated with higher tumour grade, advanced tumour-node-metastasis (TNM) stage and shorter OS. Risk score was an independent predictor for OS according to independent prognostic analysis.

We constructed a prognostic model with 6 cluster-specific marker genes (*ADH1A, ADH1B, CYP3A4, FCGBP, PABPC1, NDRG1)* in the present study. Some researchers have reported that a variation in alcohol dehydrogenase 1A (*ADH1A)* may contribute to slow alcohol metabolism, which induces increased blood acetaldehyde levels in Korean subjects [37]. In HCC patients, a high expression of *ADH1A* was associated with good survival and a less aggressive disease state [38]. Alcohol dehydrogenase 1B (*ADH1B*) is mainly known for its involvement in the major human ethanol metabolic pathway [39]. As a multifunctional enzyme, human hepatic cytochrome P-450 3A4 (*CYP3A4*) has a wide range of substrates, including commonly used drugs [40]. The expression of the Fc fragment of IgG-binding protein (*FCGBP*) is low in some tumours and high in others [41]. Low expression of *FCGBP* could be used as a crucial regulator of tumour growth factor 1 (*TGF*-1)-induced epithelial-mesenchymal transition in gallbladder cancer [42]. In contrast, high expression of *FCGBP* significantly decreases the OS of colorectal cancer patients [43]. High expression of *PABPC1* is associated with worse OS for HCC and may contribute to the progression of HCC [44]. As discussed above, *NDRG1* is a biomarker for metastasis, recurrence and a poor prognosis in HCC.

The results showed higher fractions of macrophages in the high-risk groups of the TCGA and ICGC cohorts. It has been demonstrated that increased infiltration of tumour-associated macrophages is associated with a poor prognosis in HCC patients due to their role in immune invasion [45, 46]. In addition, a high risk score was associated with impaired antitumour immunity, as indicated by the activity of the type II IFN response and type I IFN response as well as the fractions of NK cells. Therefore, it is reasonable to assume that the antitumour immunity of the high-risk group is attenuated, which may be an important reason for their poor prognosis. According to the ESTIMATE algorithm, the expression of prognostic genes was also significantly correlated with the stromal score and immune score (P < 0.05), indicating that the tumour tissue in the high-risk group was highly infiltrated by immune cells. The analysis of the expression of prognostic genes and drug sensitivity showed that all prognostic genes were correlated with the sensitivity to some chemotherapy drugs. These data demonstrated that some prognostic genes can be used as therapeutic targets to overcome drug resistance or adjuvant drug sensitivity.

In conclusion, the results of this study have demonstrated that the analysis of tumour gene expression combined with ST remarkably increases granularity when compared to bulk analysis. Tumours with negligible histological differences and various regions of the same tumour showed significant differences in the transcription profiles of tumour cells at each site. High expression of *PPIB, UGT2B15, IFI27, NDRG1, BHLHE40* and *VEGFA* may suggest the formation of satellite nodules that cannot be detected by imaging.

The study also defined a new prognostic signature consisting of six cluster-specific marker genes. We proved that the gene profile is independently associated with OS in the TCGA cohort and ICGC validation cohort and confirmed its value in the analysis of function, tumour microenvironment and drug sensitivity, providing insight for predicting the prognosis of HCC. The specific potential mechanism linking cluster-specific marker genes and tumour immunity in HCC remains unclear and is worthy of further study.

In summary, we propose that expression profiles based on spatial analysis can serve as new markers for the prediction of HCC prognosis.

## Supplementary Information

**Additional file 1.** Materials and methods.

**Additional file 2: Figure S1.** Volcano plot of significantly differentially expressed genes between tumor and peritumor. (A)Volcano plot of signifantly differentially expressed genes between tumor and peritumor in Case 1. (B)Volcano plot of signifantly differentially expressed genes between tumor and peritumor in Case 2. (C)Volcano plot of signifantly differentially expressed genes between tumor and peritumor in Case 3.

**Additional file 3: Figure S2.** Spatial comparison of marker gene expression in the other two cases. (A-B) Spatial expression of representative marker gene between tumor and peritumor clusters in case 2 and case 3. (C-D) Violin plots displaying the expression of representative marker gene identified in tumor and peritumor clusters in case 2 and case3.

**Additional file 4: Figure S3.** Enriched pathway for differentially expressed genes in tumor and peritumor. (A-B) Enriched pathway in Case 2 tissue sample. (C-E) Enriched pathway in Case 3 tissue sample.

**Additional file 5: Figure S4.** Flow chart of data collection and analysis.

**Additional file 6: Figure S5.** Constructed an 6-gene signature in the TCGA cohort. (A) LASSO coefficient expression profiles of 7 candidate genes. (B) The penalty parameter (λ) in the LASSO model was selected through ten cross-validation

**Additional file 7: Figure S6.** Survival analysis of prognostic genes in TCGA cohort.

**Additional file 8: Figure S7.** Each prognostic gene expression between HCC tissues and adjacent non-tumorous tissues in TCGA.

**Additional file 9: Figure S8.** Immunohistochemical staining for 6 cluster-specific marker genes in HCC and adjacent nontumour tissues. (A) The expression levels of ADH1A, CYP3A4 and ADH1B were lower in tumour tissues than in adjacent nontumour tissues (peritumor) and the expression levels of NDRG1 and PABPC1 were higher in tumour tissues than in adjacent nontumour tissues. (B) The expression levels of FCGBP were higher in tumour tissues than in normal liver tissues. (The Human Protein Atlas).

**Additional file 10: Table S1.** Clinicopathologic characteristics of patients.

**Additional file 11: Table S2.** Expression patterns in HCC tissues and adjacent nontumor tissues revealed in immunohistochemistry analysis.

## Data Availability

All data generated or analysed during this study are included in this published article.
