# Supplementary material for: Spatial maps of hepatocellular carcinoma transcriptomes highlight an unexplored landscape of heterogeneity and a novel gene signature for survival
Source: Cancer Cell Int. 2022 Feb 2;22:57. doi: 10.1186/s12935-021-02430-9 (PMC8812006; doi:10.1186/s12935-021-02430-9)
Supplement: Supplementary file 1 — Additional file 1. Materials and methods. [file 12935_2021_2430_MOESM1_ESM.docx]

**Supplementary materials and methods**

**Tissue Collection**

Thirty pairs of HCC samples, surgically resected during from January 2017 to October 2021, were obtained from the Tumor Tissue Bank of Tianjin Cancer Hospital. Diagnosis of HCC was verified by pathologists. All tissues were fixed in 10% formalin immediately and embedded with paraffin within 12 to 24 hours after resection. The use of these tissues was approved by the Institutional Research Committee.

**Antibody Preparation**

Primary antibodies were selected to reveal the expression of cluster-specific marker genes: ADH1A (EPR4439, 1:300 dilution, ab108203, abcam), ADH1B (OTI4F12, 1:150 dilution, TA502777S, Origene), CYP3A4 (EPR6202, 1:400 dilution, ab124921, abcam), NDRG1 (EPR5593, 1:400 dilution, ab124689, abcam) and PAPBC1 (1:500 dilution, GTX113954,GeneTex).

**Immunohistochemical staining**

Prior to immunostaining, 4μm paraffin sections were deparaffinization in xylene and rehydrated by a graded series of aqueous ethanol solutions. Endogenous peroxidase activity was blocked with 3% hydrogen peroxide in 100% methanol for 15 minutes at room temperature. Sections were washed with phosphate-buffered saline (PBS), and then pretreated with citrate buffer (pH 6.0) or Tris/EDTA buffer (pH 9.0) for 15 minutes at 95 °C in a microwave oven to expose antigens. After nonspecific binding sites were blocked by incubation in 10% normal goat serum in PBS for 20 minutes at 37 °C, sections were incubated overnight at 4 °C with the primary antibodies. The next day, sections were incubated with a compatible horse radish peroxidase (HRP)-conjugated secondary antibody for 30 minutes at 37 °C, followed by the chromogen 3,3`- diaminobenzidine for 5 to 10 minutes at room temperature. Finally, the sections were lightly counterstained with hematoxylin for minutes followed by dehydration and mounting on coverslips. For negative controls, PBS was used in place of primary antibodies. The staining systems used in this study were PicTure PV6000 (Zhongshan Chemical Co, Beijing, China) and Elivision Plus (Zhongshan Chemical Co, Beijing, China).

**Immunohistochemical Scoring**

Evaluation of sections was performed by two independent pathologists blind to clinical information. Expression of each marker was assessed semiquantitatively according to both the extension of cells stained and the intensity of immunostaining in individual tumor cell. More than 10 microscopic fields in each section were counted with about 100 tumor cells per field under light microscopy. The extent of positivity (“extent of distribution” of positive cells) was graded on the following scale: 0 for less than 10% positive cells, 1 for less than 25% positive cells, 2 for less than 50% positive cells, and 3 for more than 50% positive cells. The intensity of staining was scored on a scale of 0 to 3 as follows: 0, no appreciable staining in the tumor cells; 1, barely detectable staining in the cytoplasm and/or nucleus compared to the stromal elements; 2, readily visible brown staining; and 3, dark brown staining in tumor cells obscuring the cytoplasm and/or nucleus. The minimum score when summed (extension + intensity) was therefore, 0, and the maximum, 6. For statistical analysis, a total score of 0 to 3 were considered low expression, while scores of 4 to 6 were considered high expression.
