# Supplementary figures and images for: Spatial maps of hepatocellular carcinoma transcriptomes highlight an unexplored landscape of heterogeneity and a novel gene signature for survival
Source: Cancer Cell Int. 2022 Feb 2;22:57. doi: 10.1186/s12935-021-02430-9 (PMC8812006; doi:10.1186/s12935-021-02430-9)

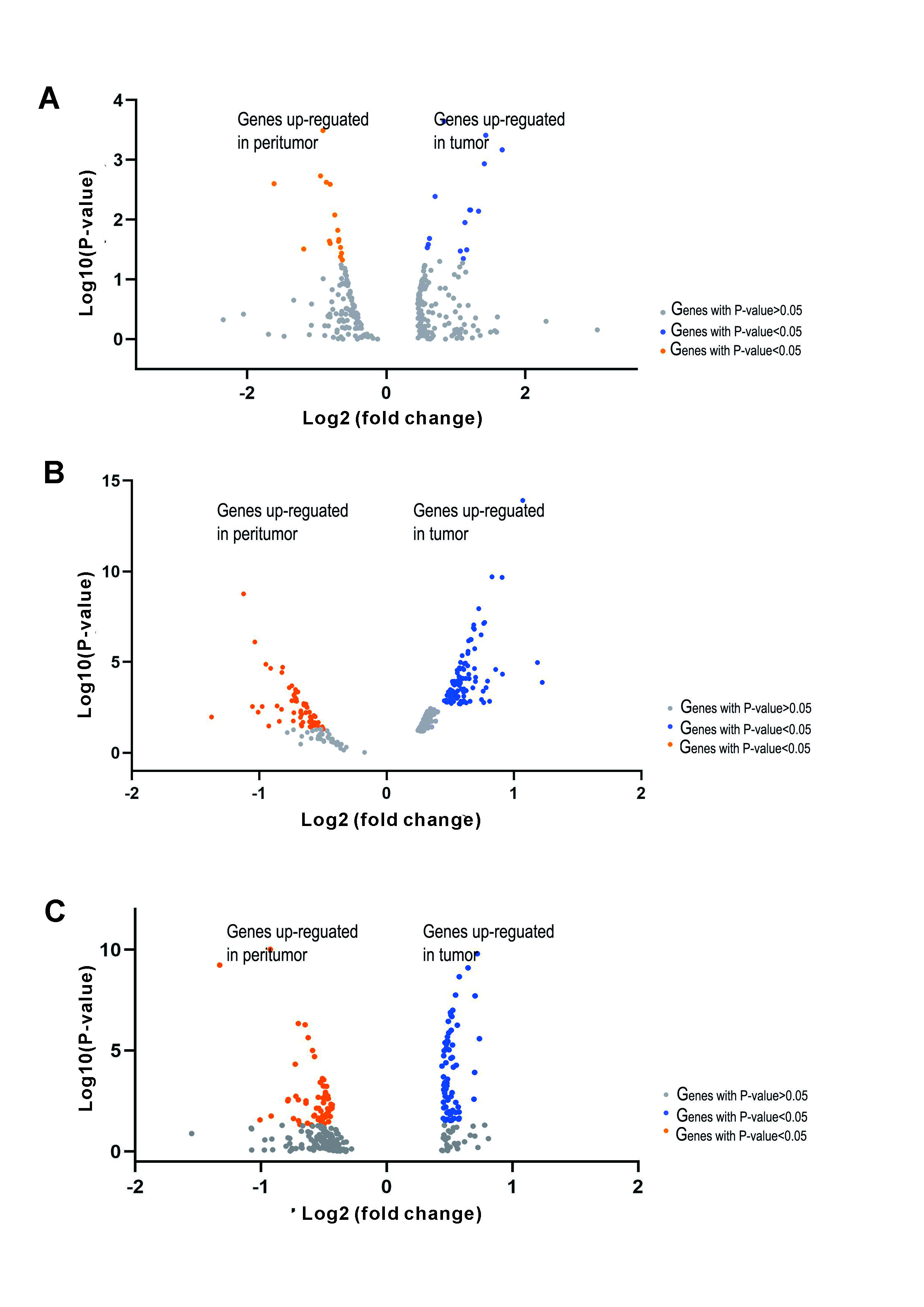

Supplement: Supplementary file 2 — Additional file 2: Figure S1. Volcano plot of significantly differentially expressed genes between tumor and peritumor. (A)Volcano plot of signifantly differentially expressed genes between tumor and peritumor in Case 1. (B)Volcano plot of signifantly differentially expressed genes between tumor and peritumor in Case 2. (C)Volcano plot of signifantly differentially expressed genes between tumor and peritumor in Case 3. [file 12935_2021_2430_MOESM2_ESM.jpg]

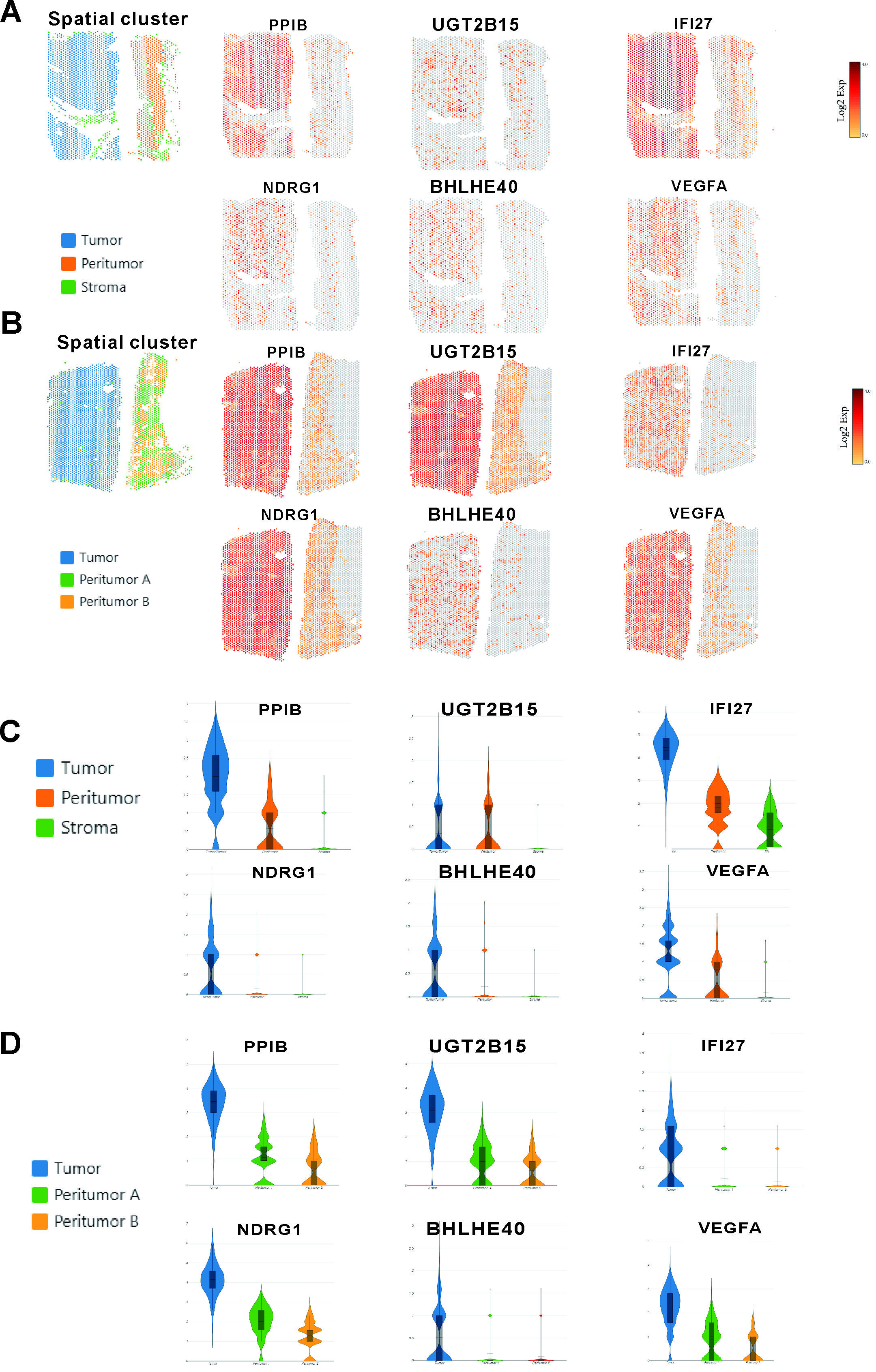

Supplement: Supplementary file 3 — Additional file 3: Figure S2. Spatial comparison of marker gene expression in the other two cases. (A-B) Spatial expression of representative marker gene between tumor and peritumor clusters in case 2 and case 3. (C-D) Violin plots displaying the expression of representative marker gene identified in tumor and peritumor clusters in case 2 and case3. [file 12935_2021_2430_MOESM3_ESM.jpg]

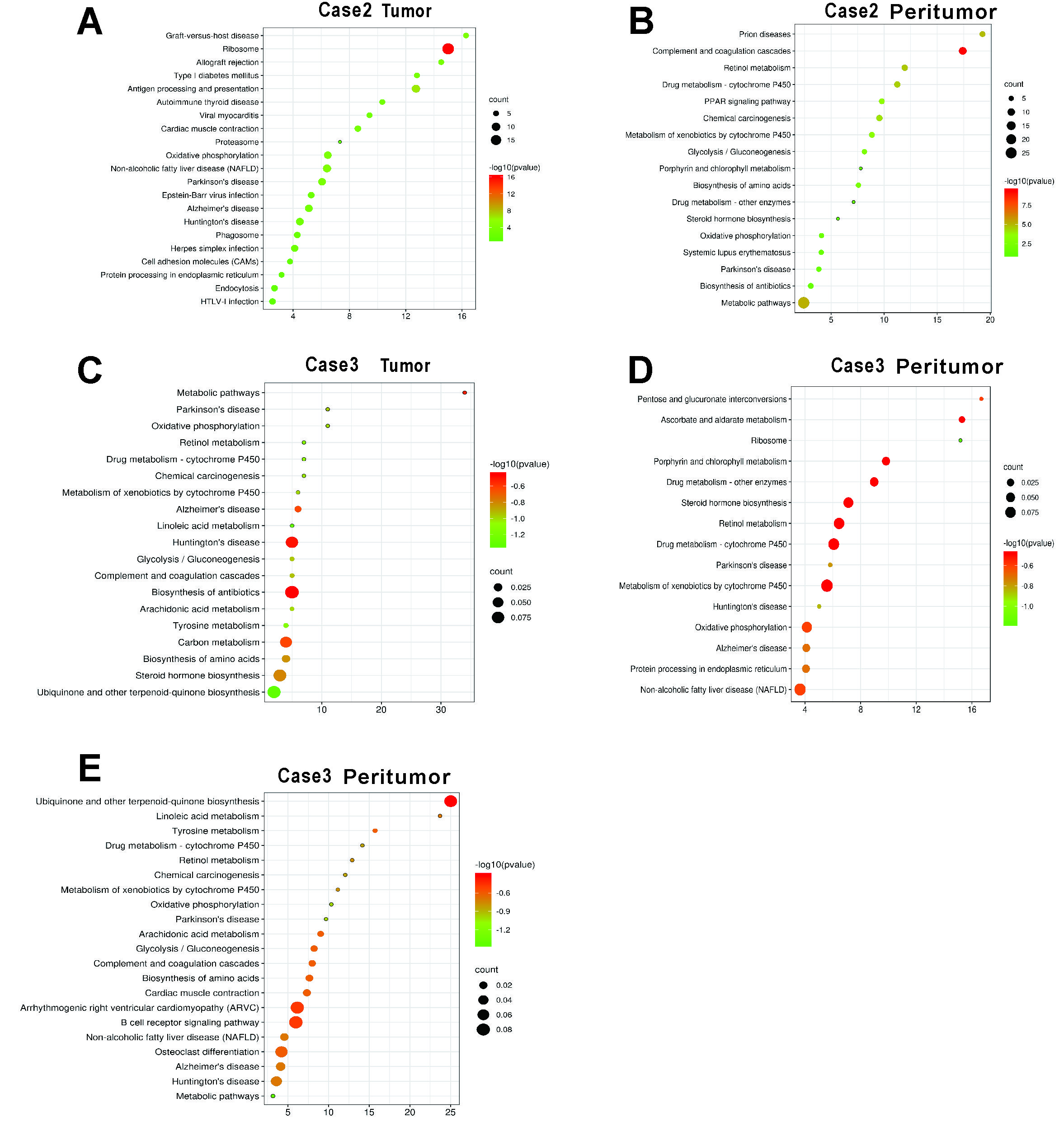

Supplement: Supplementary file 4 — Additional file 4: Figure S3. Enriched pathway for differentially expressed genes in tumor and peritumor. (A-B) Enriched pathway in Case 2 tissue sample. (C-E) Enriched pathway in Case 3 tissue sample. [file 12935_2021_2430_MOESM4_ESM.jpg]

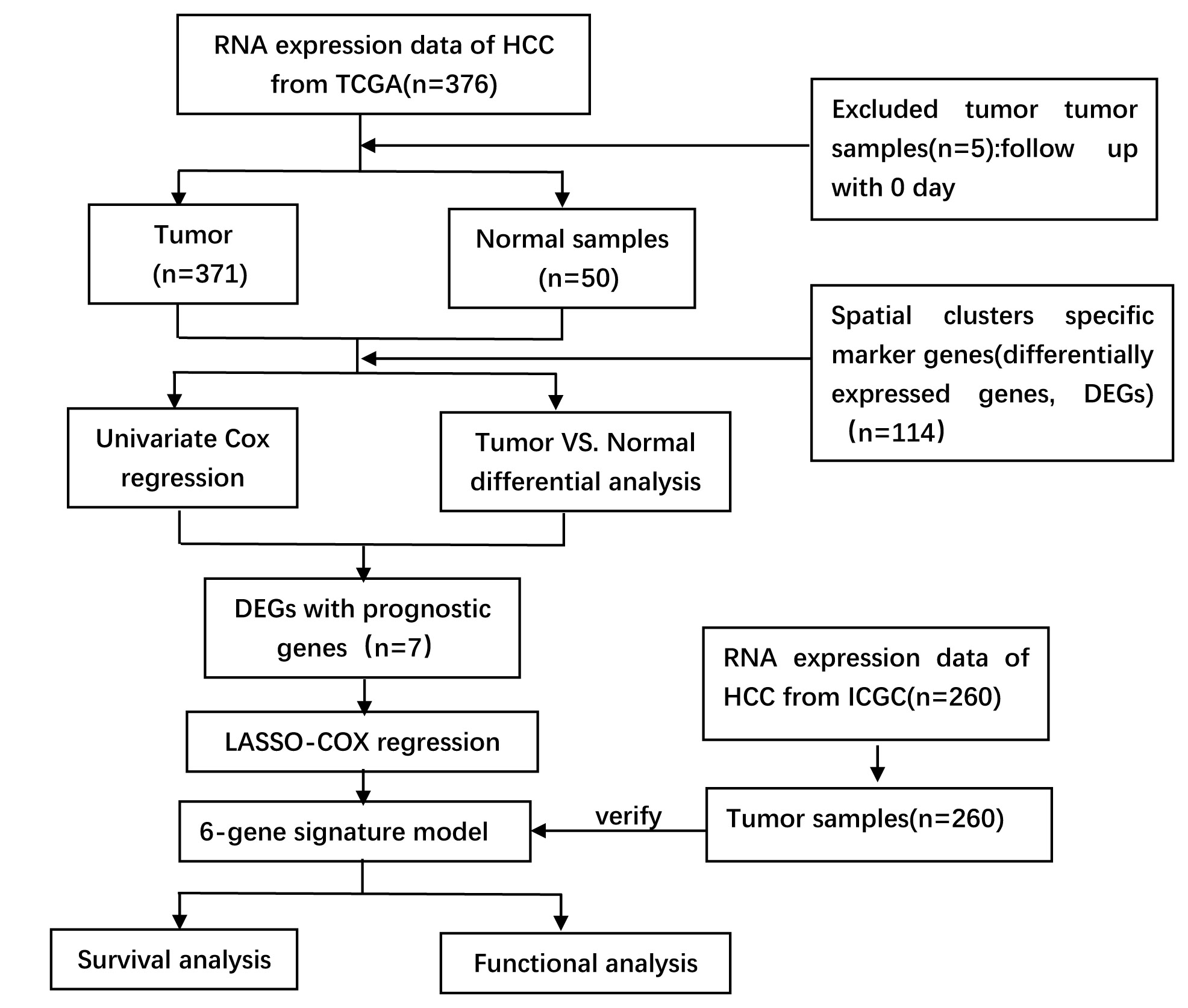

Supplement: Supplementary file 5 — Additional file 5: Figure S4. Flow chart of data collection and analysis. [file 12935_2021_2430_MOESM5_ESM.jpg]

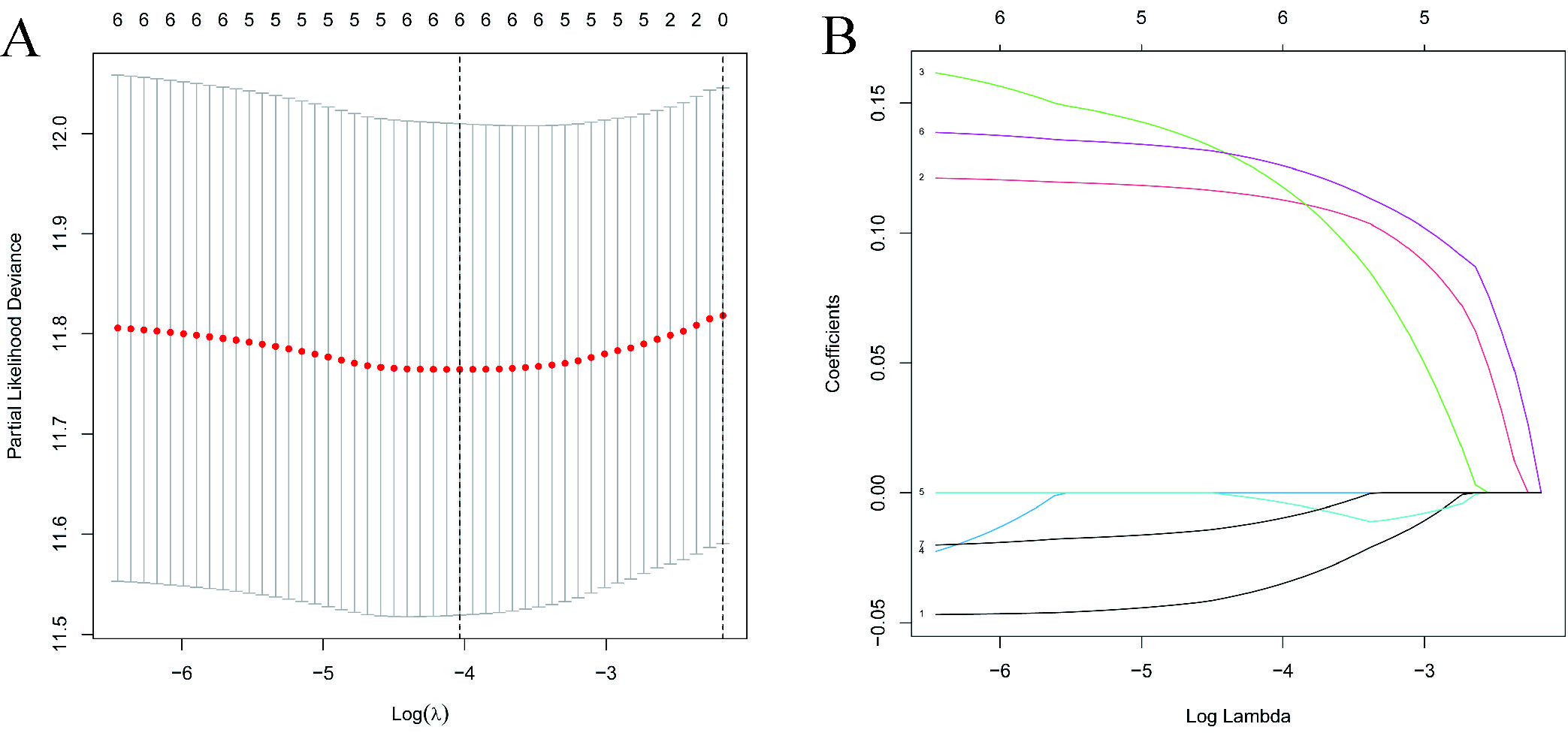

Supplement: Supplementary file 6 — Additional file 6: Figure S5. Constructed an 6-gene signature in the TCGA cohort. (A) LASSO coefficient expression profiles of 7 candidate genes. (B) The penalty parameter (λ) in the LASSO model was selected through ten cross-validation [file 12935_2021_2430_MOESM6_ESM.jpg]

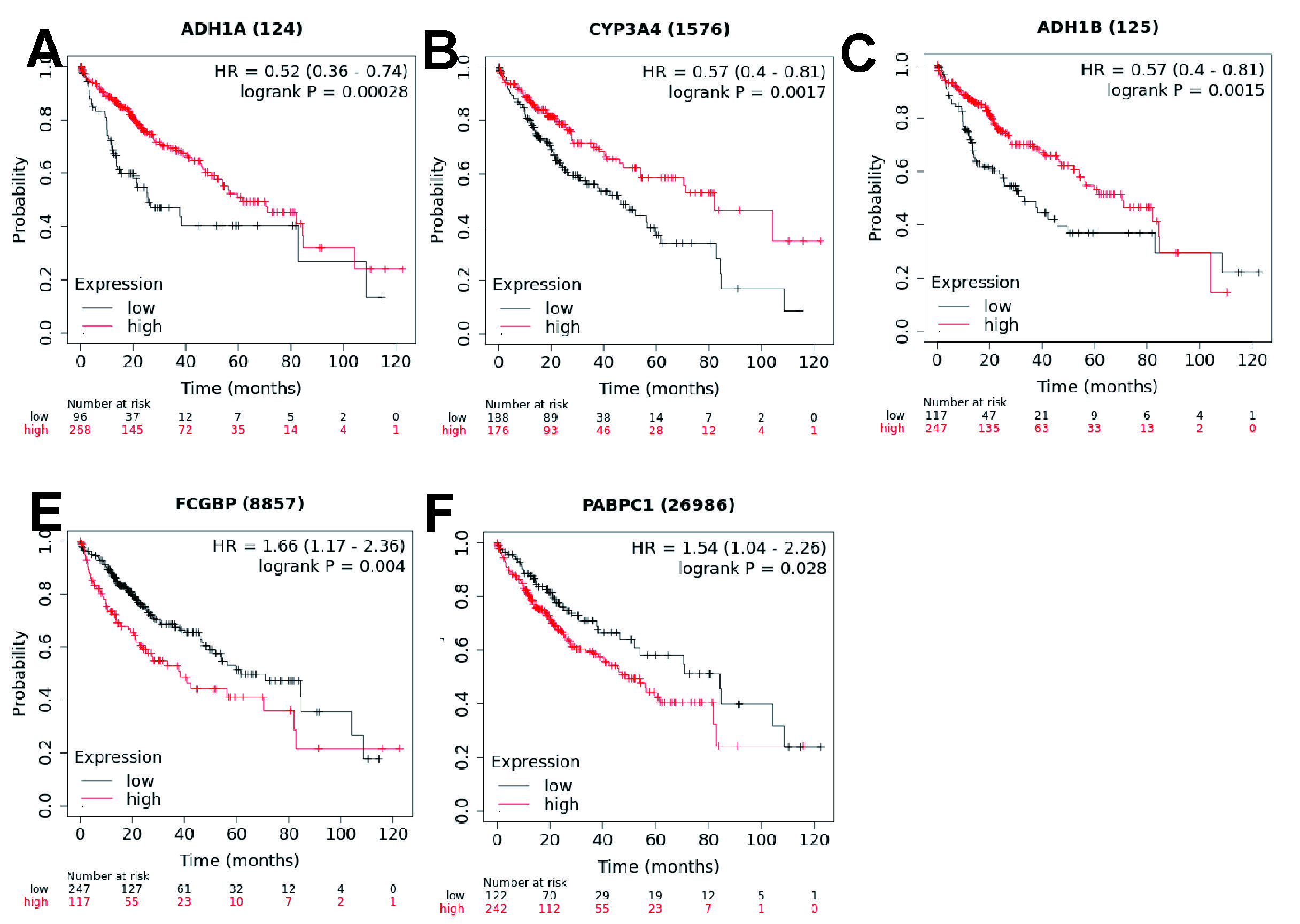

Supplement: Supplementary file 7 — Additional file 7: Figure S6. Survival analysis of prognostic genes in TCGA cohort. [file 12935_2021_2430_MOESM7_ESM.jpg]

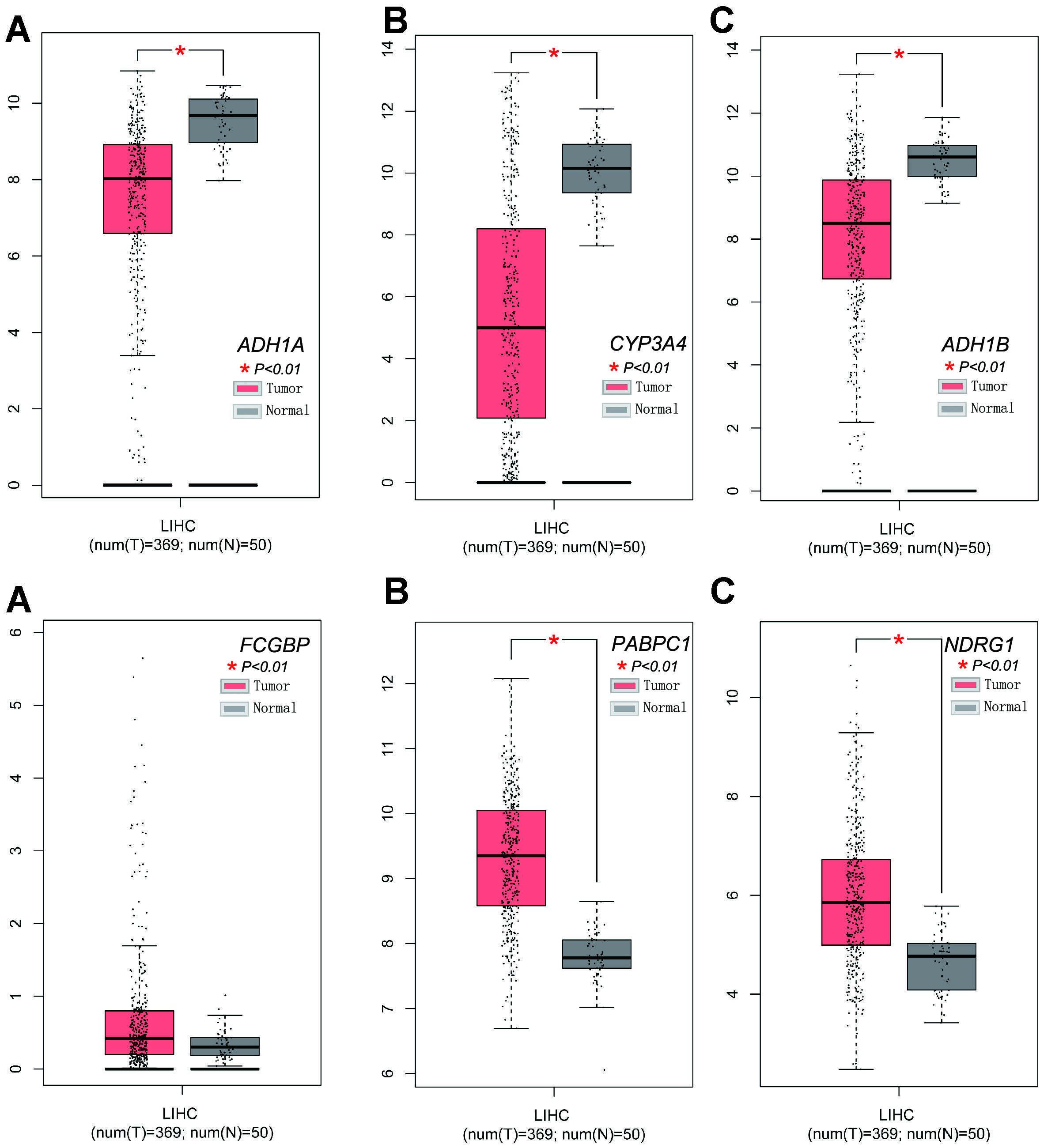

Supplement: Supplementary file 8 — Additional file 8: Figure S7. Each prognostic gene expression between HCC tissues and adjacent non-tumorous tissues in TCGA. [file 12935_2021_2430_MOESM8_ESM.jpg]

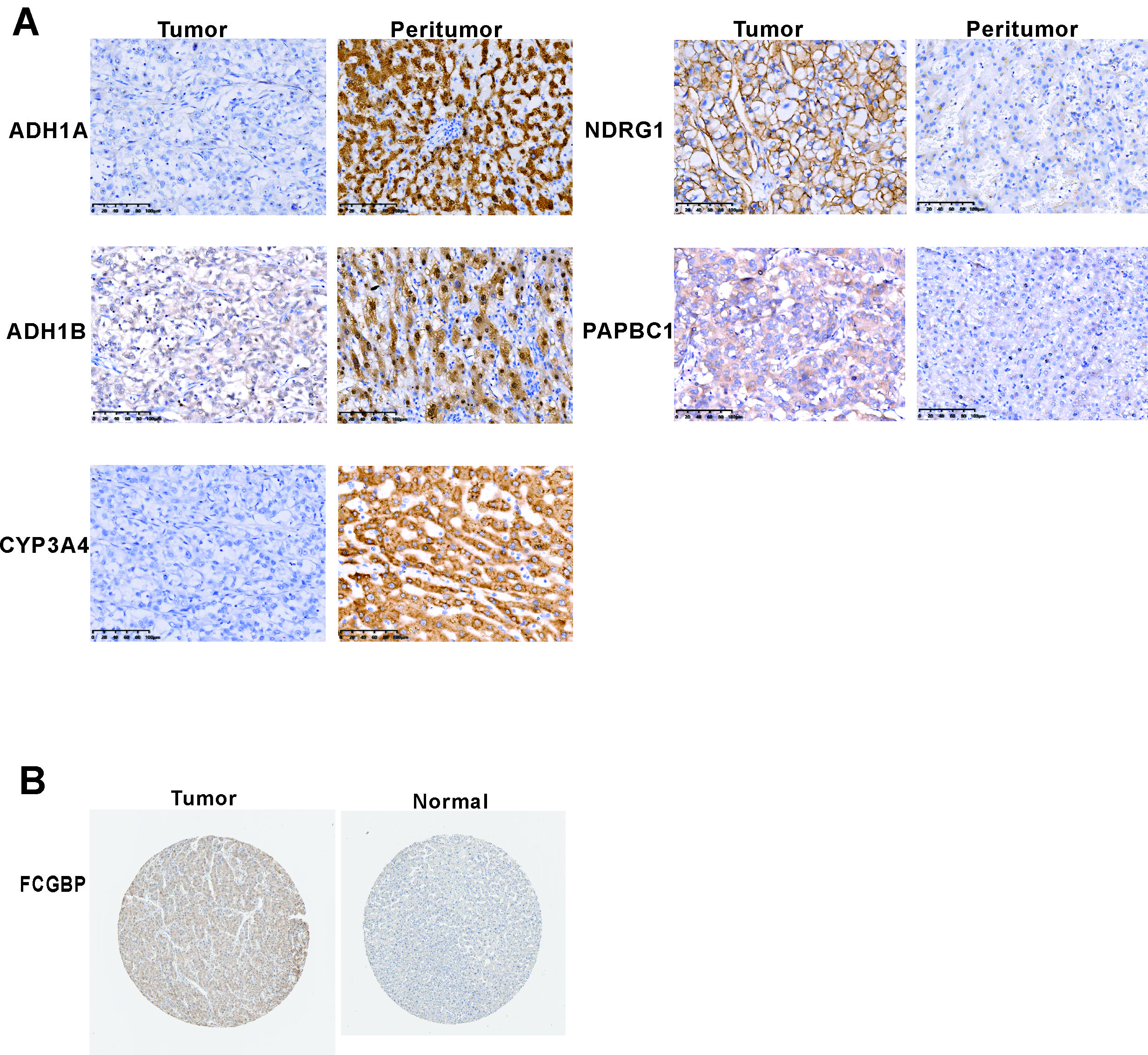

Supplement: Supplementary file 9 — Additional file 9: Figure S8. Immunohistochemical staining for 6 cluster-specific marker genes in HCC and adjacent nontumour tissues. (A) The expression levels of ADH1A, CYP3A4 and ADH1B were lower in tumour tissues than in adjacent nontumour tissues (peritumor) and the expression levels of NDRG1 and PABPC1 were higher in tumour tissues than in adjacent nontumour tissues. (B) The expression levels of FCGBP were higher in tumour tissues than in normal liver tissues. (The Human Protein Atlas). [file 12935_2021_2430_MOESM9_ESM.jpg]
