# Supplementary material for: Spatial maps of hepatocellular carcinoma transcriptomes highlight an unexplored landscape of heterogeneity and a novel gene signature for survival
Source: Cancer Cell Int. 2022 Feb 2;22:57. doi: 10.1186/s12935-021-02430-9 (PMC8812006; doi:10.1186/s12935-021-02430-9)
Supplement: Supplementary file 10 — Additional file 10: Table S1. Clinicopathologic characteristics of patients. [file 12935_2021_2430_MOESM10_ESM.docx]

**Supplementary Table 1 Clinicopathologic characteristics of patients**

| Variant | Case 1 | Case 2 | Case 3 |
| --- | --- | --- | --- |
| Age (years) | 83 | 79 | 66 |
| Gender | Female | Male | Male |
| Histological grade | Moderately | Poorly | Moderately |
| Clinical stage | T2N2M0 | T2N2M0 | T2N2M0 |
| Satellite nodules | Present | Absent | Absent |
| Cirrhosis | Absent | Present | Present |
| Chronic viral hepatitis | Absent | Present | Present |
| MVI risk factor | M2 | M1 | M2 |
