# Supplementary material for: Spatial maps of hepatocellular carcinoma transcriptomes highlight an unexplored landscape of heterogeneity and a novel gene signature for survival
Source: Cancer Cell Int. 2022 Feb 2;22:57. doi: 10.1186/s12935-021-02430-9 (PMC8812006; doi:10.1186/s12935-021-02430-9)
Supplement: Supplementary file 11 — Additional file 11: Table S2. Expression patterns in HCC tissues and adjacent nontumor tissues revealed in immunohistochemistry analysis. [file 12935_2021_2430_MOESM11_ESM.docx]

| Gene expression | Tumor tissue | |  | Adjacent nontumor tissues | | *P* |
| --- | --- | --- | --- | --- | --- | --- |
|  | Cases | Percentage |  | Cases | Percentage |  |
| ADH1A |  |  |  |  |  |  |
| Low | 11 | 32.35% |  | 0 | 0% | 0.000 |
| High | 23 | 67.65% |  | 34 | 100% |  |
| ADH1B |  |  |  |  |  |  |
| Low | 12 | 35.29% |  | 0 | 0% | 0.000 |
| High | 22 | 64.71% |  | 34 | 100% |  |
| CYP3A4 |  |  |  |  |  |  |
| Low | 21 | 61.76% |  | 0 | 0% | 0.000 |
| High | 13 | 38.24% |  | 34 | 100% |  |
| NDRG1 |  |  |  |  |  |  |
| Low | 0 | 0% |  | 19 | 55.88% | 0.000 |
| High | 34 | 100% |  | 15 | 44.12% |  |
| PABPC1 |  |  |  |  |  |  |
| Low | 3 | 8.82% |  | 7 | 20.59% | 0.039 |
| High | 31 | 91.18% |  | 27 | 79.41% |  |

Supplementary Table 2 Expression patterns in HCC tissues and adjacent nontumor tissues revealed in immunohistochemistry analysis
